# Cadmium accumulation in wheat grain: Accumulation models and soil thresholds for safe production

**DOI:** 10.1016/j.eehl.2025.100154

**Published:** 2025-05-14

**Authors:** Lu Lin, Xiaopeng Zhao, Yumeng Li, Jingbo Ling, Jinghua Ren, Qilin Liao, Dongmei Zhou, Xueyuan Gu

**Affiliations:** a State Key Laboratory of Pollution Control and Resource Reuse, School of the Environment, Nanjing University, Nanjing 210023, China; b Columbia University, New York, NY 10027, USA; c Technical Innovation Center of Ecological Monitoring & Restoration Project on Land (Arable), Geological Survey of Jiangsu, Nanjing 210018, China

**Keywords:** Cadmium, Wheat, Soil properties, Prediction model, Threshold

## Abstract

The high cadmium (Cd) accumulation ability of wheat has garnered significant attention in China. It is crucial to identify the key factors affecting Cd accumulation in wheat and to develop predictive models to derive the threshold concentration of Cd in soil for safe wheat production. A total of 311 soil–wheat paired datasets were collected from both literature and field surveys in China, in which the ranges of Cd in soil and wheat grain were 0.068–13.500 ​mg/kg and 0.006–2.190 ​mg/kg, respectively. Correlation analyses and Partial Least Squares Path Model indicated that soil Cd, soil pH, and CEC together controlled the transfer of Cd from soil to wheat. Multiple linear regression models were successfully established using soil Cd contents or bioavailable Cd (extracted by CaCl_2_ or calculated using a multi-surface speciation model), pH, and CEC as input variables to predict wheat Cd (RMSE ​= ​0.242–0.327, MAE ​= ​0.188–0.249). Furthermore, the Extreme Random Tree model (RMSE ​= ​0.221, MAE ​= ​0.165) outperformed the other seven machine learning algorithms. The thresholds for both soil total Cd and bioavailable Cd for safe wheat production were further back-calculated according to the permissible value of Cd in wheat grain, which demonstrated enhanced protection accuracy compared to the current soil quality standard. Our findings facilitate a quantitative assessment of Cd accumulation risk in wheat, offering a valuable reference for the safe production of wheat.

## Highlights

• The BCF of Cd in wheat grains is not sensitive to soil pH when pH ​> ​7.
• The factors determining Cd content in wheat grain: soil Cd ​> ​pH ​> ​CEC.
• MLR and ERT could predict the accumulation of Cd in wheat grain well.
• Various soil Cd thresholds for wheat safety were derived with enhanced precision.

## Graphical abstract

**Figure:**
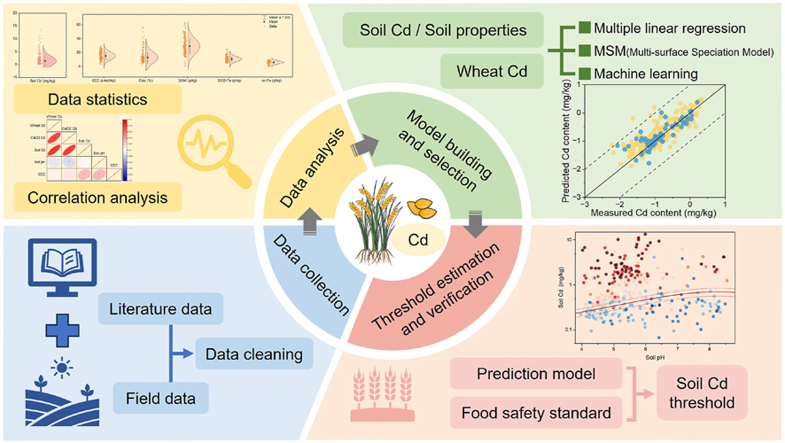

## 1 Introduction

Cadmium (Cd) is a non-essential heavy metal that is toxic to plants and animals [1]. Given its high mobility and potential for bioaccumulation in organs and subsequent serious health problems, Cd is a significant concern for human health. The accumulation of Cd in crops such as rice and wheat has been considered the predominant pathway of human (except smokers) exposure to Cd [2]. Compared with rice, wheat often exhibits a higher accumulation capacity of Cd due to its higher intra-plant Cd transport ability [3]. For example, in some rice–wheat rotation areas in China, the severity of wheat Cd exceeding the national food safety standard (GB 2762–2022) [4] is more pronounced than that of rice Cd [5,6]. In practice, it is often found that the current soil environmental quality standard of China (GB 15618–2018) [7] is more effective in protecting rice production than wheat [8,9]. To ensure the safe production of wheat, China has recently issued a more stringent recommended soil standard for Cd (GB/T 41685–2022) [10], tightening the limits from 0.3–0.6 ​mg/kg to 0.20–0.36 ​mg/kg. However, these new criteria might increase management costs and may lead to an overestimation of the actual soil risk. Considering that wheat is the primary staple crop in China, it is imperative to develop practical soil–wheat Cd transfer prediction models and to derive a more precise soil Cd threshold for the safe production of wheat.

The transfer of Cd in the soil–wheat system depends mainly on wheat varieties and soil properties [11,12]. In addition to soil Cd content, other soil properties such as soil pH, cation exchange capacity (CEC), soil organic matter (SOM), etc., may influence the accumulation of Cd in wheat [13]. Among these variables, pH is recognized as the most important one [14]. Previous models often used soil Cd content and pH as the main indices to establish the multiple regression model [13,15]. However, soil is a complex system, and the contribution of soil Cd content and pH may mask the contribution of other variables, leading to discrepancies between models and practice [16]. Therefore, other soil properties (e.g., SOM and CEC) need to be considered in the model to more accurately predict Cd accumulation in wheat [17]. The increase in the number of variables enhances the precision of the model in different soil environments, but it also elevates the complexity of models and the difficulty of application. Therefore, models must consider both aspects simultaneously.

It has been recognized that although soil total Cd is often used as a basis for soil environmental standards due to its universality, not all Cd in soil can be taken up by the plants [18,19]. In practice, bioavailable Cd is considered to be the fraction of Cd that is highly relevant for plant uptake and transfer into the food chain, which is usually the operationally defined CaCl_2_-extractable or DTPA-extractable Cd concentration. In recent years, the Multi-surface Speciation Model (MSM), a geochemical speciation model, has been reported to successfully estimate the partitioning of metal ions between the solid and liquid phases of soils [[20], [21], [22]]. Moreover, compared with chemical extraction, MSM showed better correlations with metal accumulation in various plants, such as rice [23,24], wheat [25,26], cabbage [27], and *Sedum plumbizincicola* [28]. These findings suggest that MSM-Cd possesses the potential to serve as an indicator for predicting Cd accumulation in wheat grain. Furthermore, since the MSM is based on thermodynamic equilibria, it has a good generalization ability across different soils and conditions [26], hence enabling it to derive a more robust threshold suitable for diverse soil conditions.

Previous studies have mainly used multiple linear regression (MLR); however, when faced with the introduction of multiple independent variables, MLR may increase the complexity and decrease the practicality of model construction and interpretation. Meanwhile, MLR is difficult to deal with possible multicollinearity and nonlinear relationships [29]. In recent years, the application of machine learning (ML) algorithms such as decision trees, random forests, K-Nearest Neighbor, and support vector machines in the environmental field has gained much attention [[30], [31], [32]]. These models have significant advantages over linear regression in fitting complex relationships and possess better generalization ability. Recent studies have shown the commendable predictive capability of these models in estimating metal accumulation in crops, using soil properties, nutrient elements, social economy, and geography as input features [[33], [34], [35]].

Until now, studies on quantitative modeling of Cd accumulation in wheat have mainly been at a laboratory scale, and a practical model for the field scale is still lacking. In this study, we collected a total of 358 soil–wheat data points from both field surveys and literature. We used Pearson correlation analysis and Partial Least Squares Path Modeling (PLS-PM) to identify the key contributors to wheat Cd. We also established various prediction models, including MRL and ML models, and compared the predictive effects of different soil Cd indicators, such as soil total Cd, extracted bioavailable Cd, and MSM-Cd. Finally, the soil Cd threshold for wheat safety was back-calculated based on the established models. The primary objective of this study is to establish a robust prediction model for Cd accumulation for soil–wheat systems and derive practical soil Cd thresholds for ensuring safe wheat production. This will enable the rapid prediction of wheat Cd using pre-existing soil parameters and will effectively serve the assessment and management of safe agricultural production.

## 2 Materials and methods

### 2.1 Data collection and processing

To investigate the relationship between soil properties and Cd content in wheat grain, data were collected from both the literature and our previous field surveys. Literature data were collected from publications in the Science Citation Index Expanded Database and the Chinese Science Citation Database on the Web of Science from 2015 to 2025 using the keywords “cadmium” and “wheat”. The literature was further screened based on the following criteria: (1) experiments were conducted on soils of China, either field or pot experiments; (2) wheat grain Cd content, wheat variety, soil Cd content, location of the soil, soil properties (pH, CEC, SOM, clay) and soil bioavailable Cd were given; (3) if the soil and wheat were treated in the experiments, e.g., by applying material to the soil or the leaves, only the results of the untreated group were used; (4) soil and grain samples should be completely digested with strong acids, and the Cd content should be determined by inductively coupled plasma mass spectrometry (ICP-MS), or inductively coupled plasma optical emission spectrometry (ICP-OES), or graphite furnace atomic absorption spectrometry (AAS). The soil bioavailable Cd here refers to the amount of Cd extracted by 0.01M CaCl_2_ solution [36]. A total of 92 sets of data from 41 articles were collected. Another 266 sets of soil–wheat data were obtained from field surveys in 2011, 2016, and 2018 in some rice–wheat rotation areas of Jiangsu Province, China. The data included wheat grain Cd content, soil total Cd, soil properties (pH, CEC, SOM, clay, DCB-Fe, ox-Fe), and soil bioavailable Cd. Here, DCB-Fe is the total active iron content extracted by dithionite citrate bicarbonate, and ox-Fe is the amorphous iron extracted by ammonium oxalate. The analysis methods can be found in our previous publication [37].

Among the total 358 sets of soil–wheat grain paired data, 265 sets had complete soil physical and chemical properties, while the remaining lacked iron oxide contents or clay contents, which are required for MSM calculation. We supplemented the missing iron oxide contents by quoting data from similar sites and the same soil type based on the soil location and *Soil Series of China*, and the clay contents were supplemented based on the National Soil Information Service Platform of China (http://www.soilinfo.cn).

The Cd bioconcentration factors (BCF) were then calculated according to the following equation:

(1) $$\text{BCF}={\text{Cd}}_{\text{wheat}}/{\text{Cd}}_{\text{soil}}$$

where Cd_wheat_ is the Cd content in wheat grain (mg/kg), and Cd_soil_ is the total Cd content of soil (mg/kg).

Then, a data cleaning process was performed by trimming off the samples containing missing values or outliers with extremely high or low BCF (more than 3 standard deviations) [16]. Then, the remaining 311 sets of data (46 from the literature, 265 from field surveys) were used for the next model development.

### 2.2 Model development

Two predictive model approaches for Cd accumulation in wheat grain, MLR and ML, were compared. The data set was randomly split into a training set and a test set with a ratio of 4:1, used for building and evaluating the models, respectively [30]. To ensure the stability of our results, we implemented the following approaches: 10 independent random splits and fixed random seeds. The results show that the performance of the model is similar across different data splits (Table S7). A model was finally selected from these to be shown in the study. The model-building workflow is illustrated in Fig. S1.

#### 2.2.1 Input features and output label settings

The input features were selected from soil pH, CEC (cmol/kg), clay (%), SOM (g/kg), DCB-Fe (g/kg), ox-Fe (g/kg), soil total Cd content (Cd_soil_, mg/kg), CaCl_2_ extracted Cd (${\text{Cd}}_{{\text{CaCl}}_{2}}$, mg/kg), and MSM-Cd (Cd_MSM_, mg/L) depending on the model type used. The output label is wheat Cd content (Cd_wheat_, mg/kg). In order to meet the assumption of normal distribution required for linear regression and to minimize the effect of extreme values, all non-normally distributed Cd-related data (Cd_soil_, ${\text{Cd}}_{{\text{CaCl}}_{2}}$, Cd_MSM_, and Cd_wheat_) were transformed into logarithmic form, while the others remained unchanged. In this study, the simultaneous use of Cd_soil_, ${\text{Cd}}_{{\text{CaCl}}_{2}}$, and Cd_MSM_ as input features was avoided due to their high correlations, which can lead to model overfitting and inaccurate estimates [38].

#### 2.2.2 Multiple linear regression model

The MLR equations for Cd content in wheat grain were constructed as follows:

(2) $${\text{Cd}}_{\text{wheat}}=\beta_{0}+\beta_{1}Z_{1}+\beta_{2}Z_{2}+\cdots +\beta_{n}Z_{n}$$

where Cd_wheat_ is the Cd content in wheat grain (mg/kg); β_0_ represents the constant; β_1_, β_2_, and β_n_ are the regression coefficients; Z_1_, Z_2_, and Z_n_ are the values of soil properties.

#### 2.2.3 Linear regression model for soil bioavailable Cd

Soil bioavailable Cd is influenced by soil properties and is highly correlated with Cd content in plants. Thus, it can be used as a predictor of wheat grain Cd. Besides the ${\text{Cd}}_{{\text{CaCl}}_{2}}$, the Cd_MSM_ calculated using MSM can also be regarded as an indicator of soil bioavailable Cd. The linear regression equation for Cd content in wheat grain was given:

(3) $${\text{Cd}}_{\text{wheat}}=\beta_{0}+\beta_{1}{\text{Cd}}_{{\text{CaCl}}_{2}}+\beta_{2}Z_{2}$$

or

(4) $${\text{Cd}}_{\text{wheat}}=\beta_{0}+\beta_{1}{\text{Cd}}_{\text{MSM}}+\beta_{2}Z_{2}$$

The details of the mechanism and model parameters of MSM can be found in our previous studies [21,26]. Briefly, the dissolution of Cd in soil is mainly controlled by adsorption on soil reactive surfaces (SOM, iron oxides, and clay minerals). MSM estimated the distribution of metal ions in the solid and liquid phases by considering the soil as the sum of reactive surfaces and describing the adsorption of ions on each surface using various surface complexation models (Fig. S3). The model calculation was carried out using ECOSAT 4.9 software [39] with the generalized model parameters. The solid–liquid ratios (g/L) were set at 10, 100, and 1000, and the impact of cation competition was considered [26]. The model that considered the competition of major cations and a solid–liquid ratio of 100 was finally selected (Table S1). The calculated dissolving Cd was assumed to be the bioavailable fraction of Cd in soils, i.e., Cd_MSM_. The model results with the highest correlation coefficients with Cd_wheat_ were ultimately selected for regression modeling.

#### 2.2.4 Machine learning algorithm

Four traditional ML models [i.e., Ridge, Decision Tree (DT), Support Vector Regression (SVR), and K-Nearest Neighbors (KNN)] and four ensemble models [i.e., Random Forest (RF), Extremely Randomized Trees (ERT), Gradient Boosting Decision Trees (GBDT), and Extreme Gradient Boosting (Xgboost)] were compared to identify the model with superior performance. A detailed introduction on these eight ML models can be found in Text S1. The training data set was further split into pre-training and validation sets in a ratio of 9:1, then a 10-fold cross-validation was used in the training set to avoid overfitting [40]. We referred to previous studies and performed the tuning process by manually adjusting some key hyperparameters [33]. The final model performance is verified on the test dataset. The data splitting was performed in PyCharm 2024.1.4 using the train_test_split and Kfold functions of the machine learning toolkit scikit-learn.

#### 2.2.5 Model performance evaluation

The coefficient of determination (R^2^) and two regression loss functions [root mean square error (RMSE) and mean absolute error (MAE)] are used to evaluate the performance of models. The models with the higher R^2^ and the smaller loss function values were assumed to be optimal models.

### 2.3 Thresholds for safe production

China's national food safety standard (GB 2762–2022) [4] has a guideline value of 0.1 ​mg/kg for Cd in wheat grains. The Cd threshold concentrations in soil for wheat food safety were back-calculated based on this limit and the optimal models. Briefly, the wheat Cd content was set at 0.1 ​mg/kg and the corresponding soil Cd content under different pH conditions was calculated as a threshold value. If the Cd_soil_ exceeded the threshold value, there was a risk of Cd_wheat_ exceeding 0.1 ​mg/kg. Then the derived thresholds were used to predict whether they can protect wheat safe production. The predictions were categorized into four groups: True Positive (TP, Cd_soil_ exceeds thresholds and Cd_wheat_ exceeds standards), False Negative (FN, Cd_soil_ does not exceed thresholds, but Cd_wheat_ exceeds standards), False Positive (FP, Cd_soil_ exceeds thresholds but Cd_wheat_ does not exceed standards), and True Negative (TN, Cd_soil_ does not exceed thresholds and Cd_wheat_ does not exceed standards). The accuracy (Acc) of the threshold prediction was calculated as follows:

(5) Acc%=(TP+TN)/(TP+FN+FP+TN)×100%

### 2.4 Statistical analysis

Pearson correlation analysis was conducted using Origin 2023b to reveal the correlation between soil Cd content, soil properties, and wheat grain Cd content with a significance level of *p* ​< ​0.05 (two-tailed). PLS-PM analysis was performed using the “plspm” package in the R statistical environment. MLR was performed in SPSS 25 software.

## 3 Results and discussion

### 3.1 Soil properties and Cd content in soil–wheat systems

**Fig. 1:**
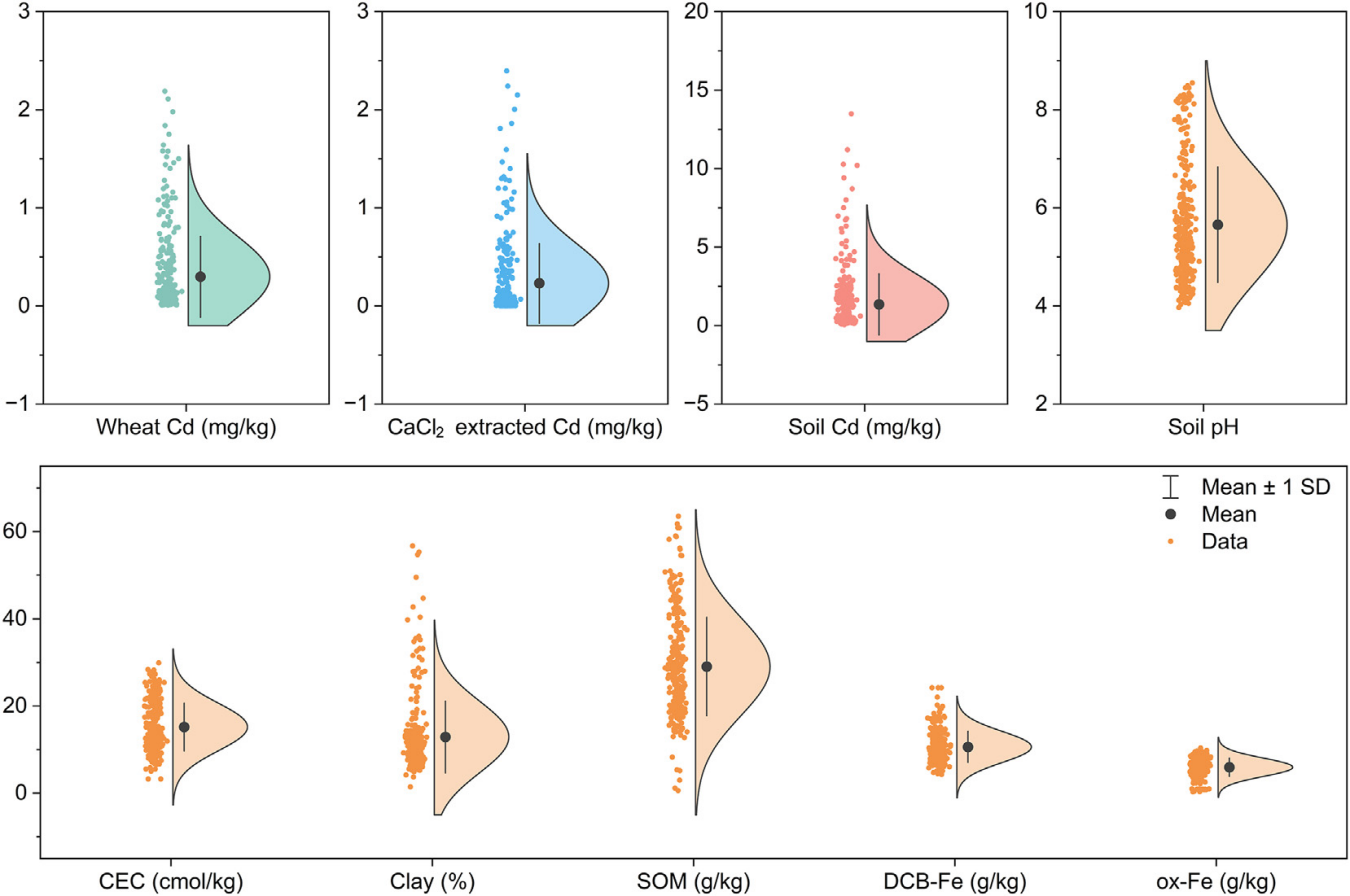
Distribution of wheat grain Cd content, soil bioavailable Cd, soil Cd content, and soil properties in the dataset (n ​= ​311). DCB-Fe is the total active iron content extracted by dithionite citrate bicarbonate, and ox-Fe is the amorphous iron extracted by ammonium oxalate.

The collected dataset on wheat and soil properties covers the major wheat-producing regions of China [41,42] (Fig. S2), including Henan, Jiangsu, Anhui, and Jiangxi. The statistical distribution of soil properties was summarized using box plots in Fig. 1. Soil pH ranged from 3.97 to 8.55 with a mean value of 5.65, indicating that the dataset contained a wide range of soils from acidic to alkaline. Other soil properties also contain a wide range of data, such as soil CEC (3.25–29.90 ​cmol/kg), clay (1.46%–56.70%), SOM (0.605–63.510 ​g/kg), DCB-Fe (4.30–24.20 ​g/kg), and ox-Fe (0.31–10.37 ​g/kg), which are all normally distributed and suggest a good representation of the data set.

The Cd contents in soil and wheat grain are in the range of 0.068–13.500 ​mg/kg and 0.006–2.190 ​mg/kg, with average values of 1.329 and 0.298 ​mg/kg, respectively. According to the guideline values for soil contamination of agricultural land (GB 15618–2018) and food safety limit for wheat grain (GB 2762–2022), 67.85% of the soil and 51.13% of the wheat grain in the dataset exceeded the standards. The high proportion of over-limit results observed here can be attributed to publication biases, as the reported studies have predominantly focused on contaminated agricultural land. The range of ${\text{Cd}}_{{\text{CaCl}}_{2}}$ content is 0.032 μg/kg–2.397 ​mg/kg, with an average value of 0.230 ​mg/kg. The BCF of wheat falls within the range of 0.01–0.72, with an average value of 0.26, which is consistent with previous results in laboratory experiments and field surveys where mean BCF values were reported as 0.25 and 0.21, respectively [5,43]. This high BCF of wheat indicates a greater potential of Cd accumulation compared to maize and rice because the latter two have been reported with mean BCF values of 0.16 and 0.15 in two meta-analysis studies where China was the main data source [3,16]. The high BCF of wheat highlights the necessity to establish special prediction models for Cd transfer in soil–wheat systems.

### 3.2 Critical factors of Cd transfer from soil to wheat grain

**Fig. 2:**
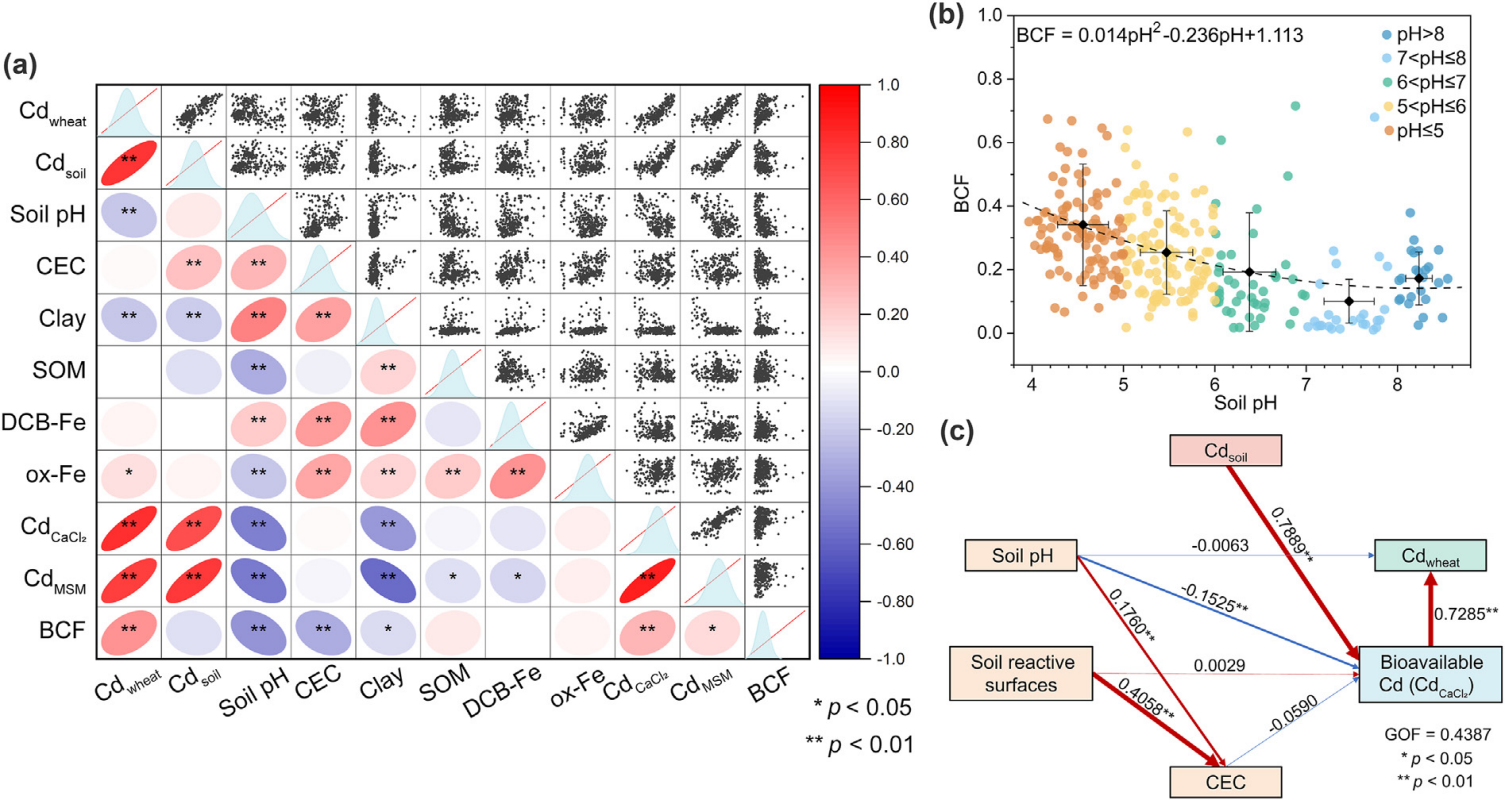
(a) Pearson correlations between soil properties, Cd_soil_, ${\mathrm{Cd}}_{{\mathrm{CaCl}}_{2}}$, Cd_MSM,_ and Cd_wheat_. Cd-related data were transformed into logarithmic form. Pearson correlations are shown in the lower left triangular matrix; light blue plots are frequency distributions of features; scatter plots are displayed in the upper right triangular matrix. (b) Relationship between the bioconcentration factor (BCF) of Cd and soil pH. The dashed line is the fitted equation. (c) Partial Least Squares Path Modeling (PLS-PM) between the soil properties, Cd_soil_, ${\mathrm{Cd}}_{{\mathrm{CaCl}}_{2}}$, and Cd_wheat_. The soil's reactive surface included organic matter, iron oxides, and clay minerals. Goodness-of-fit (GOF ​= ​0.4387) is used to measure the effectiveness of the model fit. Paths are shown as red lines if positive or as blue lines if negative; the widths of the lines and numbers represent the total effect between the latent variables in the model.

There are many factors that might affect Cd accumulation in wheat grains besides Cd_soil_ being the primary one. Therefore, Pearson correlations were performed to identify the critical factors in determining the Cd_wheat_ (Fig. 2a). The results showed that Cd_soil_ significantly positively correlated with Cd_wheat_ (*r* ​= ​0.796, *p* ​< ​0.01), because soil is the most important source of cation uptake by plants. Soil pH has a negative correlation with Cd_wheat_ (Fig. 2a), which aligns with previous reports [15,23]. However, the correlation coefficient between them was relatively low at −0.209 (*p* ​< ​0.01), possibly due to the wide range of Cd_soil_ in our dataset. To mitigate the influence of Cd_soil_, we further examined the correlation between soil pH and BCF, an index reflecting the transferability of Cd from soil to wheat grain. A significantly negative correlation was observed (r ​= ​−0.402, *p* ​< ​0.01) (Fig. 2a and b), indicating that Cd is readily taken up by plants under acidic conditions but is immobilized in alkaline soils [44,45]. In addition, both clay and ox-Fe exhibited a significant correlation with Cd_wheat_, while the former showed a negative correlation (r ​= ​−0.217, *p* ​< ​0.01) and the latter showed a positive correlation (r ​= ​0.125, *p* ​< ​0.05). Previous studies have demonstrated that CEC and SOM of soil are important factors influencing the transportation of Cd in soil and the transfer of Cd from soil to plants [8,11,46]. However, no significant correlation between SOM or CEC with Cd_wheat_ was observed in this study. The reason may be similar to soil pH, where the strong influence of Cd_soil_ masks the contribution of CEC and SOM to Cd transfer [16]. We did observe a negative correlation of BCF with CEC (r ​= ​−0.308, *p* ​< ​0.01), indicating that soils with high CEC tend to retain more Cd. However, no correlation was found between BCF and SOM, indicating that SOM played a minor role compared with CEC and soil pH in the Cd transfer in the soil–wheat system.

CaCl_2_-extracted Cd and MSM-Cd were used to represent the bioavailable fractions of Cd in soils, which are believed to be more closely related to plant uptake of Cd because they already incorporate the effect of soil properties [47,48]. Our findings demonstrated that the ${\text{Cd}}_{{\text{CaCl}}_{2}}$ and Cd_MSM_ are also strongly positively correlated with Cd_wheat_ like Cd_soil_ (Fig. 2a). The correlation coefficients have an order of ${\text{Cd}}_{{\text{CaCl}}_{2}}$ ​> ​Cd_soil_ ​> ​Cd_MSM_. Based on the correlation coefficients, ${\text{Cd}}_{{\text{CaCl}}_{2}}$ may be better used as an input feature for predicting Cd_wheat_. Conversely, the weaker correlation observed for Cd_MSM_ than that of Cd_soil_ may be because 14.7% of the data lacked the soil properties required for accurate MSM calculation.

To further identify the explanatory power of the Cd and soil properties to Cd_wheat_, the PLS-PM model was established to quantitatively assess the impact pathway [49]. The model consists of nine manifest variables (namely Cd_soil_, ${\text{Cd}}_{{\text{CaCl}}_{2}}$, Cd_wheat_, soil pH, clay, SOM, DCB-Fe, ox-Fe, and CEC), where clay, SOM, DCB-Fe, and ox-Fe were combined as the latent variable “soil reactive surfaces”, and were designated as reflective indicators. This means that the changes in these four variables are caused by changes in “soil reactive surfaces”. A path model matrix was used to describe possible associations between latent variables. Then the model estimated the path relationships between the variables. The estimated direct effect (λ_d_), indirect effect (λ_i_), and total effect (λ_t_) of the variables are shown in Table S2. Results showed that Cd_soil_ positively and significantly contributed to ${\text{Cd}}_{{\text{CaCl}}_{2}}$ (λ_d_ ​= ​0.7889) (Fig. 2c); soil pH had a negative effect on ${\text{Cd}}_{{\text{CaCl}}_{2}}$ (λ_d_ ​= ​− 0.1525) and a positive effect on CEC (λ_d_ ​= ​0.1760). Together, the four soil reactive surfaces had no significant impact on ${\text{Cd}}_{{\text{CaCl}}_{2}}$. However, they posed a positive and significant contribution to CEC (λ_d_ ​= ​0.4058). CEC negatively contributed to ${\text{Cd}}_{{\text{CaCl}}_{2}}$ (λ_d_ ​= ​− 0.0590). Finally, ${\text{Cd}}_{{\text{CaCl}}_{2}}$ contributed 0.7285 to Cd_wheat_. The results showed that soil pH and CEC were the most important factors controlling the content of bioavailable Cd, and ultimately, bioavailable Cd directly affects Cd_wheat_. Combining the results of Pearson correlation analysis and PLS-PM, we conclude that Cd_soil_, soil pH, and CEC are the key factors affecting Cd_wheat_.

### 3.3 Model building and model performance comparison

**Fig. 3:**
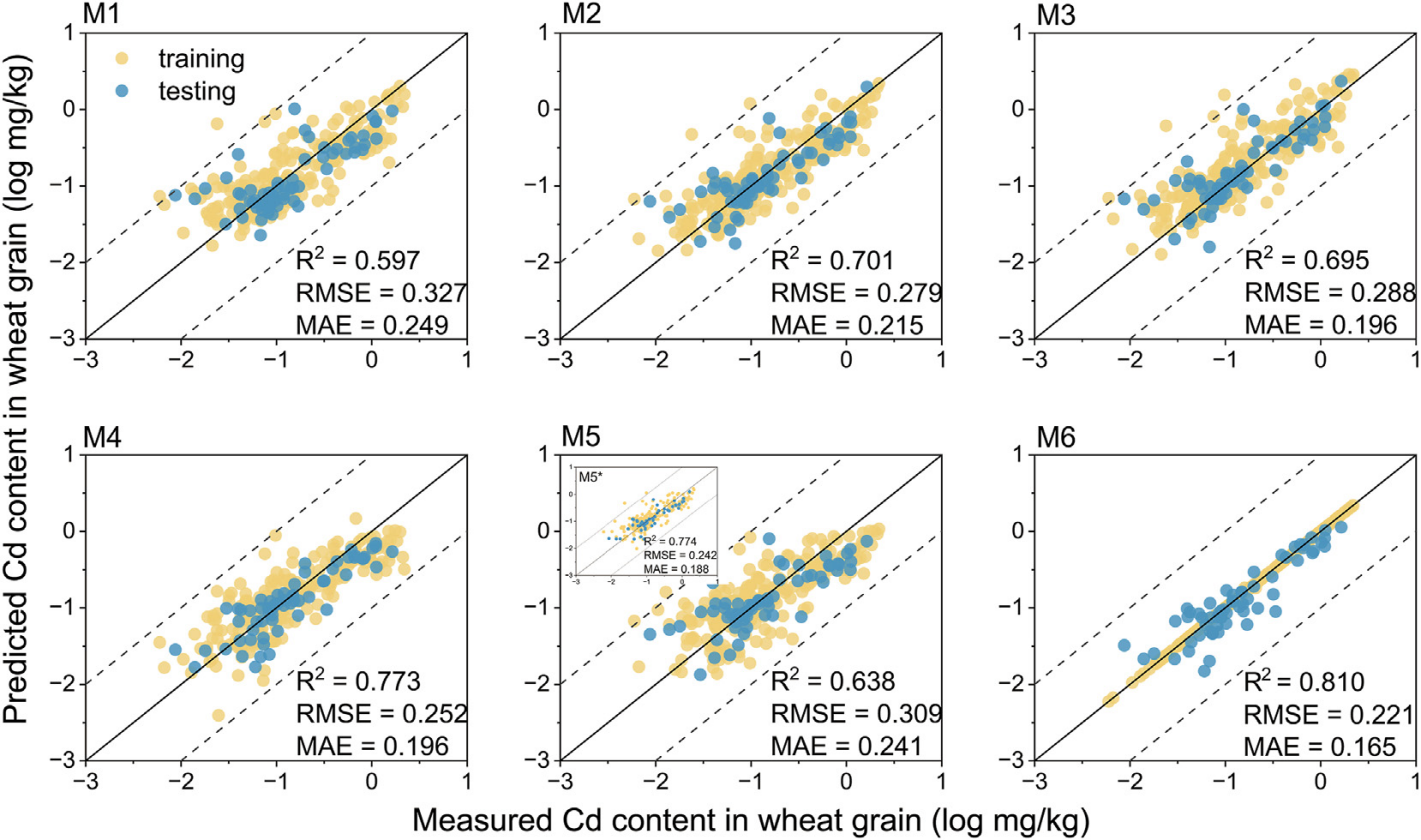
Comparison of predicted and measured Cd content in wheat grain (n ​= ​331 in M1–M6 and n ​= ​265 in M5∗). M1–M6 correspond to the model numbers in Table 1. The solid lines are the 1:1 lines, and the dashed lines are the 1:1 ​± ​1 log_10_ unit lines. The yellow dots represent the training set, and the blue dots represent the test set. R^2^, RMSE, and MAE represent the model performance in the test set.

The current section focuses on the development of predictive models for Cd bioaccumulation in wheat grain using different approaches, followed by an evaluation to identify the superior ones with the test dataset (Fig. 3).

The MLR equations were first derived based on various soil properties (model M1–M3 in Table 1). Results showed that Cd_soil_ alone was able to explain 64.3% of the variance (M1). Consistent with previous studies [50], the inclusion of soil pH and CEC increased the explanatory power to 73.0% of the variance (M2). Moreover, M2 (RMSE ​= ​0.279, MAE ​= ​0.215) exhibited superior predictive performance compared to M1 (RMSE ​= ​0.327, MAE ​= ​0.249) on the test dataset (Table 1). The coefficients of soil Cd content, pH, and CEC in the M2 regression equation were 0.995, −0.118, and −0.010, respectively. The magnitude of these absolute values indicates that the effects of these variables on Cd uptake by wheat grain were progressively diminishing, which aligns with the PLS-PM analysis. The addition of other variables, like clay, SOM, DCB-Fe, and ox-Fe, did not significantly improve the model performance (Table S3).

**Table 1:** Models of Cd content in wheat grain and model performance.

Model	Equation/Algorithm	n^b^	R^2^ (training)	R^2^ (test)	RMSE (test)	MAE (test)
M1	logCd_wheat_ ​= ​0.906logCd_soil_ ​− ​0.718	311	0.643	0.597	0.327	0.249
M2	logCd_wheat_ ​= ​0.955logCd_soil_ ​− ​0.118pH ​− ​0.010CEC ​+ ​0.114	311	0.730	0.701	0.279	0.215
M3	Cd_wheat_ ​= ​BCF ​× ​Cd_soil_ BCF ​= ​0.014pH^2^ ​− ​0.236pH ​+ ​1.113	311	0.724	0.695	0.288	0.196
M4	logCd_wheat_ ​= ​0.509log${\text{Cd}}_{{\text{CaCl}}_{2}}$ ​+ ​0.104pH ​− ​0.768	311	0.690	0.773	0.252	0.196
M5	logCd_wheat_ ​= ​0.572logCd_MSM_ ​+ ​0.106pH ​− ​0.257	311	0.607	0.638	0.309	0.241
M5∗^a^	logCd_wheat_ ​= ​0.690logCd_MSM_ ​+ ​0.020pH ​+ ​0.385	265	0.823	0.774	0.242	0.188
M6	ERT	311	1.000	0.810	0.221	0.165

Units: CEC (cmol/kg), Cd_wheat_ (mg/kg), Cd_soil_ (mg/kg), ${\mathrm{Cd}}_{{\mathrm{CaCl}}_{2}}$ (mg/kg), and Cd_MSM_ (mg/L based on a 1:10 solid–liquid ratio).

a M5∗: Model built and tested using data with complete physical and chemical properties of the soil.

b The whole dataset was randomly divided into a 4:1 ratio for the training and test datasets.

A significant and negative correlation was identified between the BCF of Cd and soil pH, which could be well described by a quadratic equation (Fig. 2b). Therefore, the wheat grain Cd can be obtained by the BCF and Cd_soil_, i.e., the model M3 in Table 1, which well predicted the Cd_wheat_ in the test dataset (RMSE ​= ​0.288, MAE ​= ​0.196). The interesting correlation between the BCF and soil pH suggested that the BCF did not decrease linearly with increasing soil pH. Instead, it first declined at pH ​≤ ​7 and then stabilized at pH ​> ​7. This pattern indicated that wheat may still maintain a certain accumulation capability of Cd in alkaline soil. It is probably because the lack of nutrients in alkaline soils may result in the co-mobilization of nutrients and Cd by wheat roots [51], or the low molecular weight organic acids and dissolved organic matter in the soil porewater increased the bioavailability of Cd [52,53]. This poses special challenges for soil remediation as alkaline amendments (e.g., adding lime) are often not as effective in alkaline soils as in acidic soils [54].

The bioavailable Cd in soil, i.e., the ${\text{Cd}}_{{\text{CaCl}}_{2}}$ and Cd_MSM_, was also used to establish the predictive models. Since the bioavailable Cd pool already integrated the influence of soil properties and soil total Cd, no other variables were added to the equations. However, since PLS-PM showed that pH still has some direct effects on Cd_wheat_ besides the bioavailable Cd, soil pH was also included to establish the MLR. Results showed that the performance of ${\text{Cd}}_{{\text{CaCl}}_{2}}$ (M4, RMSE ​= ​0.252, MAE ​= ​0.196) on the test dataset was superior to that of M2 and M3 (Fig. 3). The performance of M5 based on dissolved Cd calculated by a geochemical model of MSM (RMSE ​= ​0.309, MAE ​= ​0.241) was relatively weaker than that of M2–M4. This result is not in line with our previous findings. For example [37], found a higher correlation between MSM-Cd and wheat-Cd than CaCl_2_–Cd in a field survey. The reason may be mainly because some soil properties required by the MSM model were missing in this dataset (such as DCB-Fe and ox-Fe), leading to deviations in calculation [55]. When the data with missing soil properties were removed from the total dataset, the subsequent re-fitted model (M5∗, RMSE ​= ​0.242, MAE ​= ​0.188) exhibited superior predictive performance compared to M1–M4. This indicates that MSM has a high accuracy in predicting Cd content in wheat grains when soil properties are complete. Moreover, since the MSM model in this study used “generic” rather than “specific” model parameters, the predictive ability of the MSM could potentially be further improved by employing more specific parameters [56]. Nevertheless, during field surveys, it is not feasible and economical to measure specific parameters for each soil sample. The existing soil databases in China and generic MSM model parameters have demonstrated sufficient capability in predicting Cd_wheat_ with a slightly reduced accuracy. Hence, practical application requires striking a balance between accuracy and convenience.

In addition to the upper simple algebraic algorithms, eight ML algorithms were also trained to predict Cd_wheat_ based on the eight input features. The performance of the eight ML models is summarized in Table S3, and the model parameters are shown in Table S4. Results showed that ERT was found to be the best one on the test dataset, with RMSE and MAE being 0.221 and 0.165, respectively (Table 1).

As shown in Table S3, ridge regression, a linear regression technique used to solve the multicollinearity problem, showed good predictive results in this study. The ridge regression model achieved an R^2^ of 0.803 on the test set, and the RMSE and MAE were reduced to 0.225 and 0.167, which were better than the prediction results of the multiple linear regression model. The SVR algorithm showed poor predictive performance, probably due to the inappropriate choice of kernel function and regularization parameter. The principle of KNN is to calculate the average of the K-nearest neighbors as the predicted value, which can only predict the training samples within the coverage range, and the test samples with true values exceeding the range cannot be accurately predicted. Therefore, KNN was not effective in this study. DT is a commonly used machine learning algorithm. Unfortunately, DT (R^2^ ​= ​0.526) did not perform as well as the MLR on the test set, probably because of overfitting.

Ensemble models (RF, ERT, GBRT, and Xgboost) were found to have superior performance compared to other algorithms. The number of trees in the forest was identified as a key hyperparameter. Although increasing the number of trees usually improves model performance, it was found that the number of trees beyond 100 did not result in much improvement in model performance, so the final parameter was chosen to be 100 (Table S5). Among these models, ERT (M6) showed the best performance in predicting Cd content in wheat, achieving the maximum R^2^ and minimum RMSE and MAE on the test set (R^2^ ​= ​0.810, RMSE ​= ​0.221, MAE ​= ​0.165), which was consistent with the performance of Cd content in rice in another study [33].

Figure S4 shows the ranking and weighting of the features corresponding to the optimal ML model ERT. The most important feature of Cd uptake by wheat was soil Cd, which contributed significantly more to the model than the other variables, accounting for 66.542%. The remaining features have an order of soil pH (10.975%), clay (5.017%), ox-Fe (5.004%), CEC (4.863%), DCB-Fe (4.011%), and SOM (3.588%). Soil Cd and soil pH are still the most important features influencing Cd uptake in wheat, which is consistent with the results in Section 3.2. In an ML study of Cd accumulation features in wheat from Henan, China, the two most important features were soil phosphorus (P) and zinc (Zn), while CEC and pH were ranked fifth and eighth, respectively [57]. This may be because the data was all from Henan Province, where almost all of the soil is alkaline, so the role of soil pH was masked. However, this study also illustrates the importance of soil nutrients such as P and Zn, and consideration of nutrients may lead to further enhancement of the model. There are still areas for improvement. For example, features such as wheat variety and growth period might have an influence on heavy metal accumulation [34], but they are missing in current models due to difficulties in data collection. On the other hand, although ML algorithms are effective for predicting wheat grain Cd, they cannot calculate backward, making it difficult to set a reasonable threshold. The content of wheat grain must be predicted before it can be determined whether the samples are likely to exceed the permissible value. This limits the applicability of machine learning in threshold calculations.

### 3.4 Thresholds for wheat safe production

**Fig. 4:**
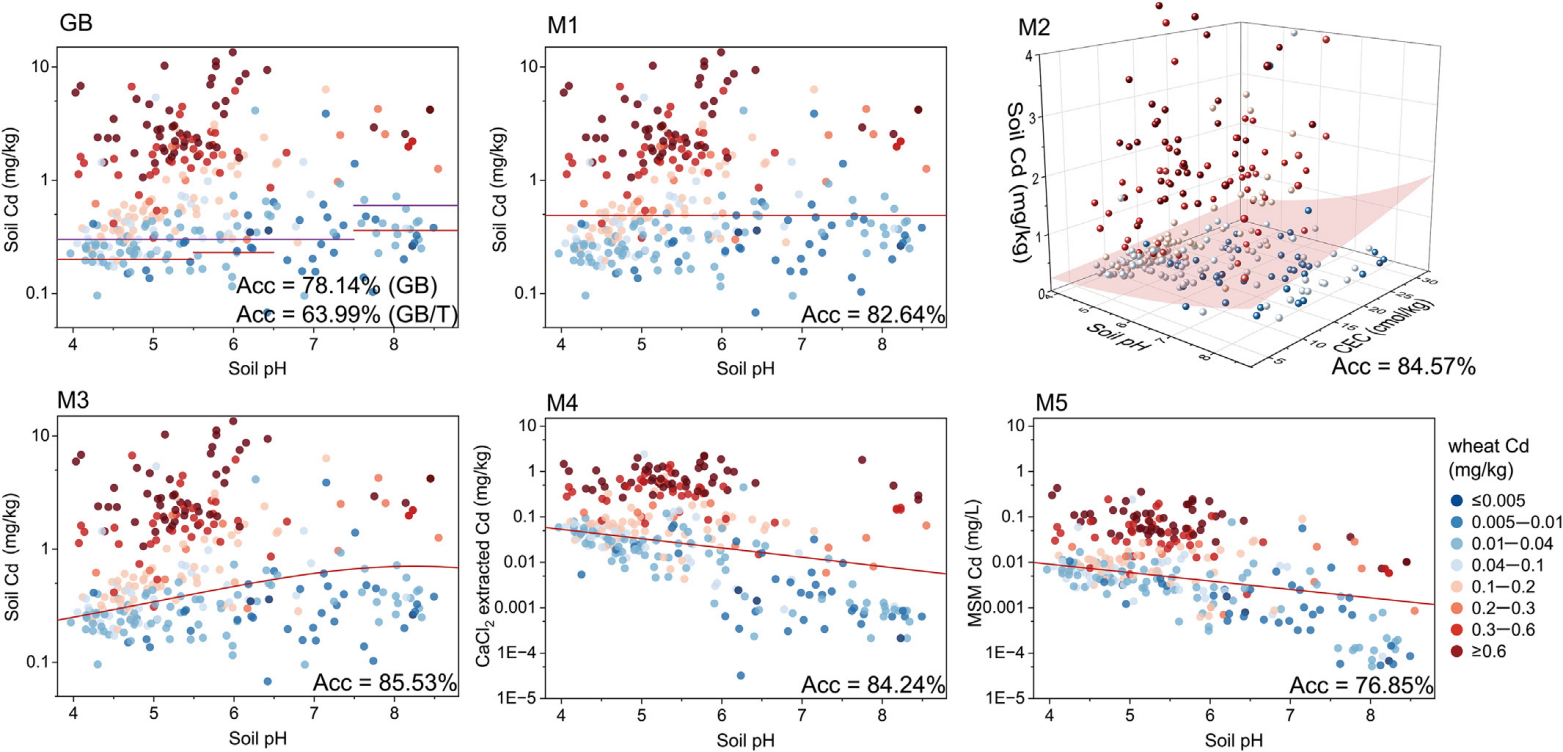
Current and derived soil Cd thresholds for wheat safe production. GB and GB/T refer to current China soil quality standards (SQS) GB 15618–2018 and GB/T 41685–2022, respectively, and M1–M5 are the derived thresholds based on MLR models in Table 1. The color of the dots (n ​= ​311) represents the level of Cd contents in wheat, where the red dots represent samples that exceeded the food quality standard of China (FQS, GB 2762–2022) and the blue ones are samples below the FQS. The red solid lines (GB, M1, M3, M4 and M5) and red surface (M2) represent the threshold values.

There are currently two existing soil quality standards (SQS) for wheat production in China: a mandatory one (GB 15618–2018) and a recommended one (GB/T 41685–2022). Both of them provide stage-specific thresholds of total soil Cd concentrations based on soil pH ranges (Table 2). To derive more accurate soil Cd thresholds for wheat, we back-calculated the soil Cd in equations M1–M5 by setting the wheat grain Cd level as 0.1 ​mg/kg, which corresponds to the maximum permissible value for Cd in wheat grain according to the food quality standard of China (FQS, GB 2762–2022). The obtained Cd thresholds based on models M1–M5 are also presented in Table 2, where thresholds derived from models M1–M3 correspond to soil total Cd contents, while thresholds derived from models M4 and M5 correspond to bioavailable Cd extracted by CaCl_2_ or calculated by MSM, respectively. Based on these thresholds, we further made judgments on whether Cd_wheat_ exceeded the FQS for all the data, and the accuracy of the results is expressed as Acc% (Eq. 4) and presented in Fig. 4 and Fig. S5.

**Table 2:** Current standards and derived threshold of soil Cd content for safe wheat production based on models in Table 1.

Model	Threshold of soil Cd content (mg/kg)			
	pH ​≤ ​5.5	5.5<pH ​≤ ​6.5	6.5<pH ​≤ ​7.5	pH ​> ​7.5
Total soil Cd content				
GB 15618–2018	0.30	0.30	0.30	0.60
GB/T 41685–2022	0.20	0.23	0.30	0.36
M1	0.488			
M2^a^	0.307	0.461	0.605	0.795
M3^b^	0.252	0.401	0.541	0.669
Bioavailable soil Cd content				
${\text{Cd}}_{{\text{CaCl}}_{2}}$ (M4)^b^	0.053	0.026	0.016	0.010
Cd_MSM_ (M5)^b^^,^^c^	0.009	0.005	0.003	0.002

a The CEC required for M2 back-calculation is based on the average value in the dataset.

b The actual threshold is successive values as a function of pH (Fig. 4); here we only list the representative values for comparison.

c The unit is mg/L based on a 1:10 solid–liquid ratio.

The soil Cd thresholds, as back-calculated by model M1–M3, were slightly higher than current standards, indicating that current standards are more stringent, especially for high pH soils. The correct recognition rates based on current standards were 78.14% (GB 15618–2018) and 63.99% (GB/T 41685–2022), while thresholds derived from M1–M3 exhibited correct recognition percentages of 82.64%, 84.57%, and 85.53%, respectively. This discrepancy can be attributed to the misclassification of a significant portion of non-exceeding samples as exceeding samples (i.e., the false positive) according to GB 15618–2018 and GB/T 41685–2022 (Fig. S5). GB 15618–2018 is a soil environmental quality standard for agricultural land based on multiple protection targets (e.g., rice, wheat, maize, etc.), while GB/T 41685–2022 is a standard for a specific crop, wheat. The latter is more stringent in its designation to protect food safety, but it may also lead to “overprotection”, resulting in higher administrative costs and waste of resources.

In the process of soil contaminant modeling and threshold derivation, biotic and abiotic factors can affect the rationality of thresholds. Therefore, the ability of wheat varieties to accumulate heavy metals should be taken into account when establishing thresholds for the soil environment. For example, researchers derived safe thresholds for cadmium transfer for two rice varieties with high and low Cd accumulation, and the results showed significant differences in soil thresholds for these two varieties [9]. In terms of abiotic factors, many standards have been established that do not always take into account more soil properties. Our study suggests that it is not sufficient to consider soil pH alone (Fig. 4) and that the combination of soil pH and CEC is the major controlling factor in the transfer of Cd from soil to wheat and should be considered when setting standards. This suggests the need for more practical environmental thresholds and improved scientific rigor and effectiveness of thresholds.

The majority of countries have established SQS based on the total concentration of heavy metals in the soils. In some areas, due to the soil parent materials and soil pedogenesis (e.g., carbonate-rich soils and high organic matter soils), although the soil Cd content is high, the bioavailability of Cd in the soil is low [58,59]. Therefore, the application of the threshold based on the total Cd content is not always appropriate [60], and some studies have used DTPA-extracted Cd as thresholds [61]. Our study also estimated the threshold for wheat food safety based on ${\text{Cd}}_{{\text{CaCl}}_{2}}$ and Cd_MSM_ concentrations (Table 2 and Fig. 4). The thresholds derived from M4 and M5 reached accuracies of 84.24% and 76.85%, respectively. Considering its wide applicability and simplicity of extraction, we proposed that soil bioavailable Cd is more suitable as the soil Cd threshold for wheat food safety. In addition to the CaCl_2_ extraction method, alternative methods such as EDTA, DTPA, and weak acid might also be employed to determine the thresholds. Furthermore, the distinctive advantage of MSM is that it can utilize existing survey data on soil Cd content and soil properties to calculate Cd_MSM_ without the need for additional field surveys, chemical extraction, and determination efforts. Fig. S7 illustrates how to select appropriate models and thresholds for field applications.

## 4 Conclusion

Wheat has a strong ability to accumulate Cd from the soil, as evidenced by the high BCF. Correlation analysis indicated that soil Cd content was the primary factor influencing the Cd transfer from soil to wheat grain, partly masking the important role of soil pH and CEC. Based on soil Cd and soil properties (i.e., soil pH and CEC) or soil bioavailable Cd, MLR models were developed to quantitatively predict Cd accumulation in wheat grain. The models that incorporated Cd_soil_, pH, and CEC as variables (M2, RMSE ​= ​0.279, MAE ​= ​0.215) and the model that considered Cd_MSM_ as a variable (M5∗, RMSE ​= ​0.242, MAE ​= ​0.188) demonstrated satisfactory reliability in their ability to predict Cd_wheat_. The ERT model (M6, RMSE ​= ​0.221, MAE ​= ​0.165) outperformed the other seven ML algorithms in predicting Cd_wheat_. In addition, these models can be used to estimate soil Cd thresholds for wheat-safe production. We proposed threshold values based on both total and bioaccessible soil Cd concentrations. The estimated Cd threshold concentrations can determine more accurately whether wheat can be produced safely in soils. We recommend adding other soil properties (e.g., CEC) to the standard to more rationally delineate the threshold range or using soil bioavailable Cd as the basis for delineating thresholds to minimize “overprotection” and reduce management and remediation costs. Our results could provide several methods for predicting Cd in wheat grain and a reliable basis for developing soil limit values for the safe production of wheat and the risk control of Cd contamination in arable soils in China.

## CRediT authorship contribution statement

**Lu Lin:** Writing – original draft, Methodology, Investigation, Data curation. **Xiaopeng Zhao:** Software, Methodology. **Yumeng Li:** Software. **Jingbo Ling:** Visualization. **Jinghua Ren:** Data curation. **Qilin Liao:** Data curation. **Dongmei Zhou:** Writing – review & editing, Project administration. **Xueyuan Gu:** Writing – review & editing, Funding acquisition, Conceptualization.

## Declaration of competing interests

The authors declare that they have no known competing financial interests or personal relationships that could have appeared to influence the work reported in this paper.
