# Supplementary material for: Cadmium accumulation in wheat grain: Accumulation models and soil thresholds for safe production
Source: Eco Environ Health. 2025 May 14;4(2):100154. doi: 10.1016/j.eehl.2025.100154 (PMC12152888; doi:10.1016/j.eehl.2025.100154)
Supplement: Multimedia component 1 [file mmc1.docx]

**Supporting Information for**

Cadmium accumulation in wheat grain: enrichment models and soil thresholds for safe production

Lu Lin ^a^, Xiaopeng Zhao ^a^, Yumeng Li ^b^, Jingbo Ling ^a^,

Jinghua Ren ^c^, Qilin Liao ^c^, Dongmei Zhou ^a^, Xueyuan Gu ^a,^*

^a^ *State Key Laboratory of Pollution Control and Resource Reuse, School of the Environment, Nanjing University, Nanjing, China*

^b^ *Columbia University in the City of New York, New York, NY 10027, USA*

^c^ *Technical Innovation Center of Ecological Monitoring & Restoration Project on Land (arable), Geological Survey of Jiangsu, Nanjing, China*

* Corresponding author


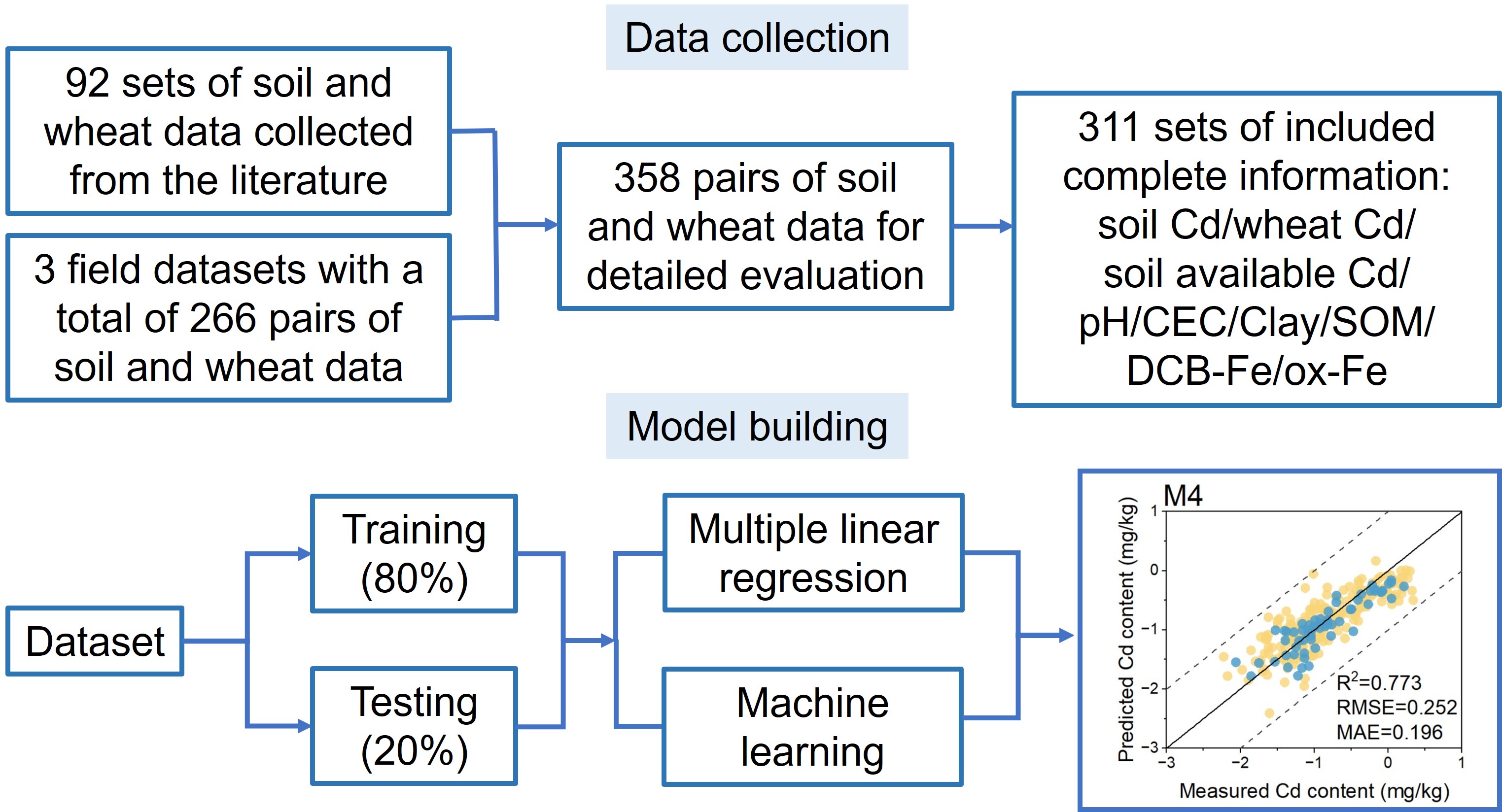


**Figure S1.** Data collection and model-building scheme

**
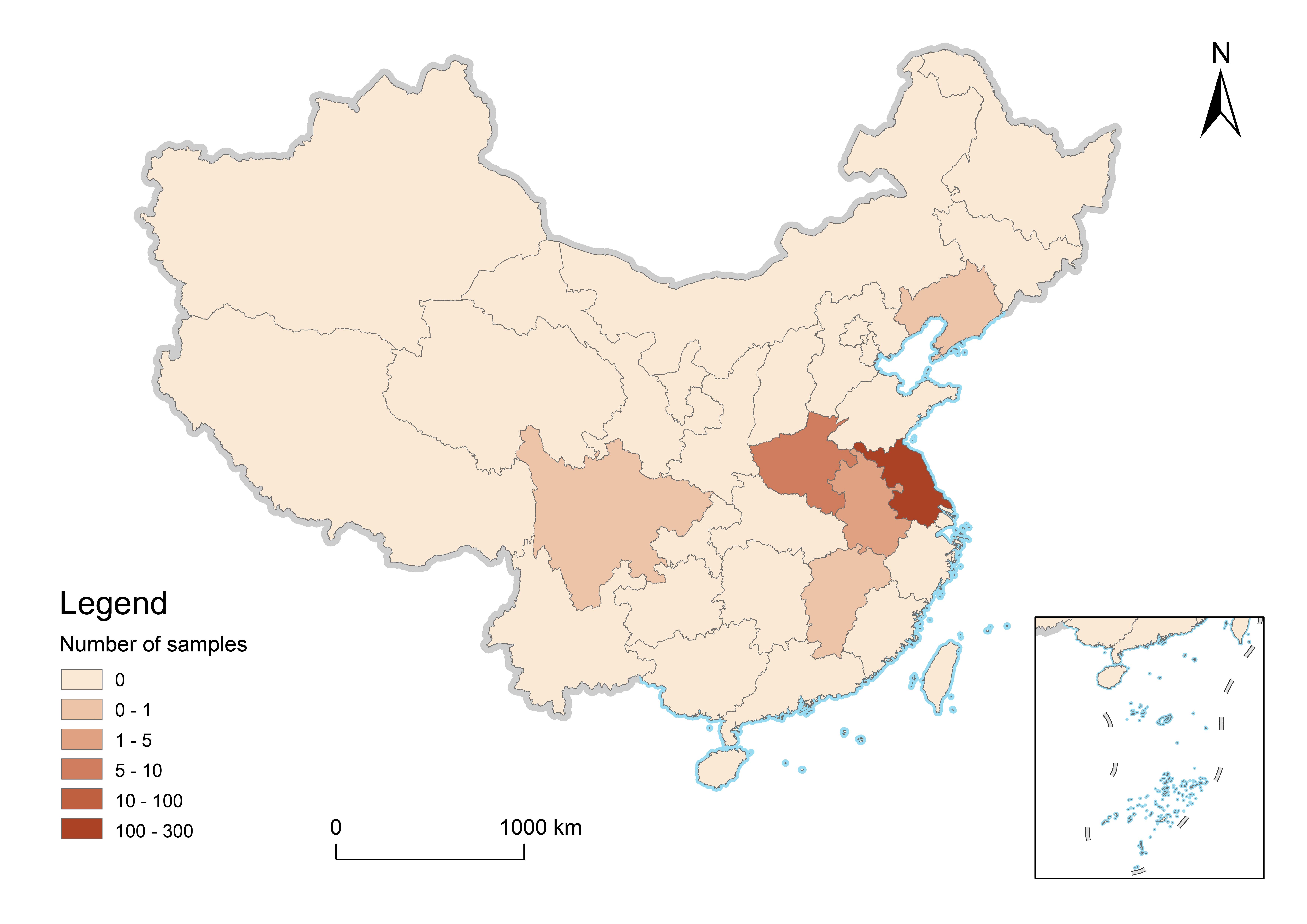
**

**Figure S2**. Map of soil sample locations (n=311).


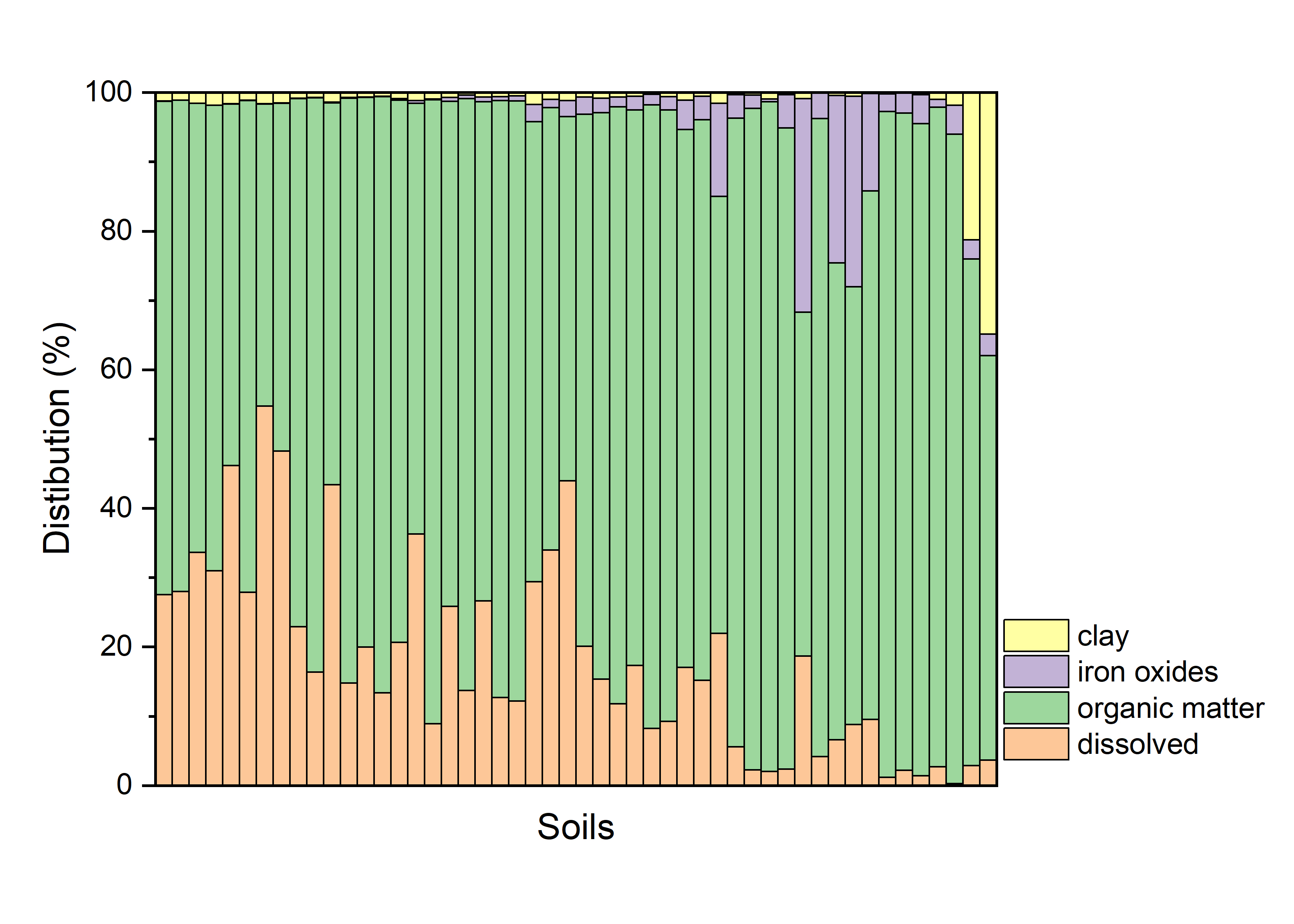


**Figure S3**. The distribution of Cd onto soil components in the first 50 samples in the training set, as predicted by MSM. The soils were ordered according to soil pH from low to high.


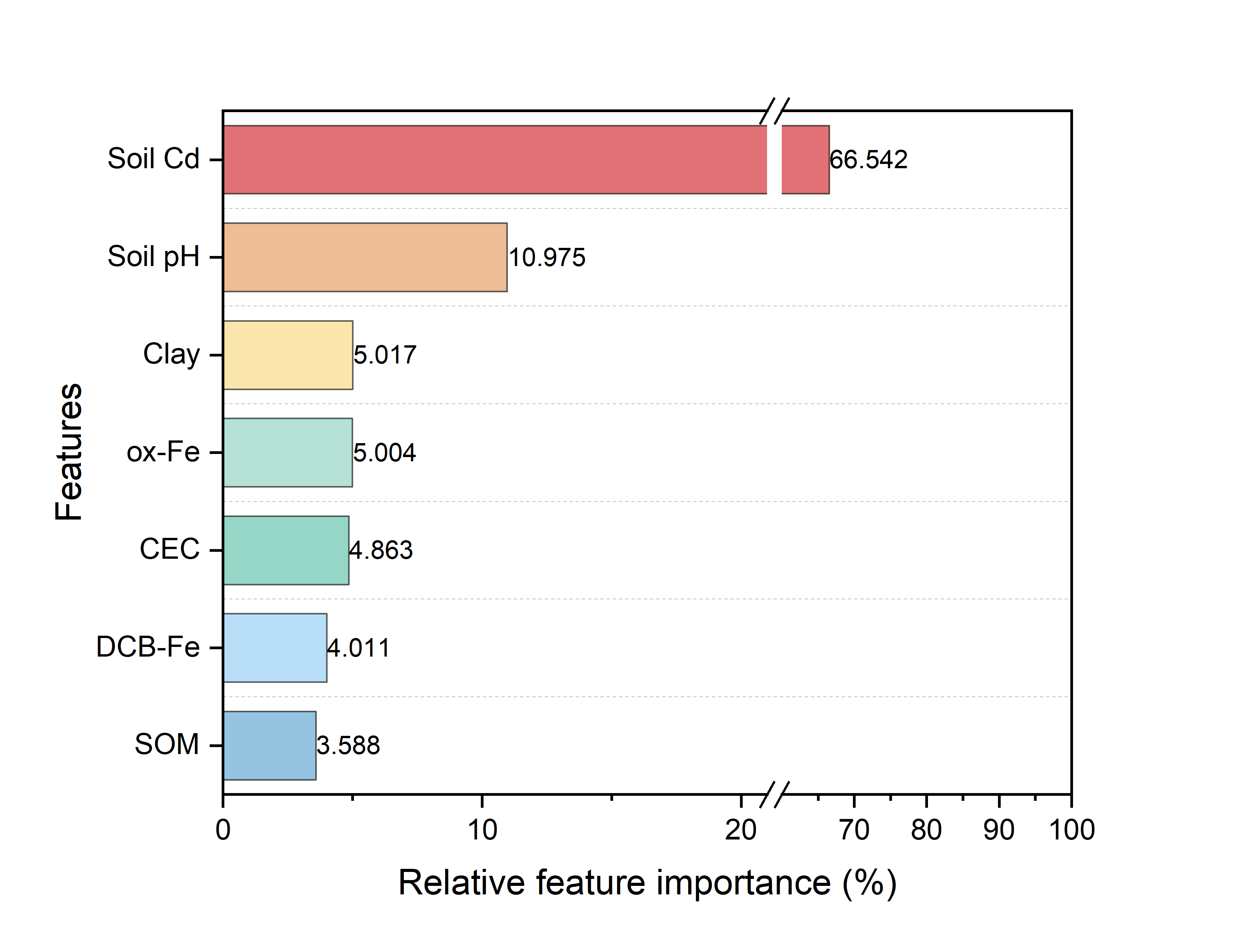


**Figure S4**. Importance of input features in predicting wheat grain Cd content using ERT.


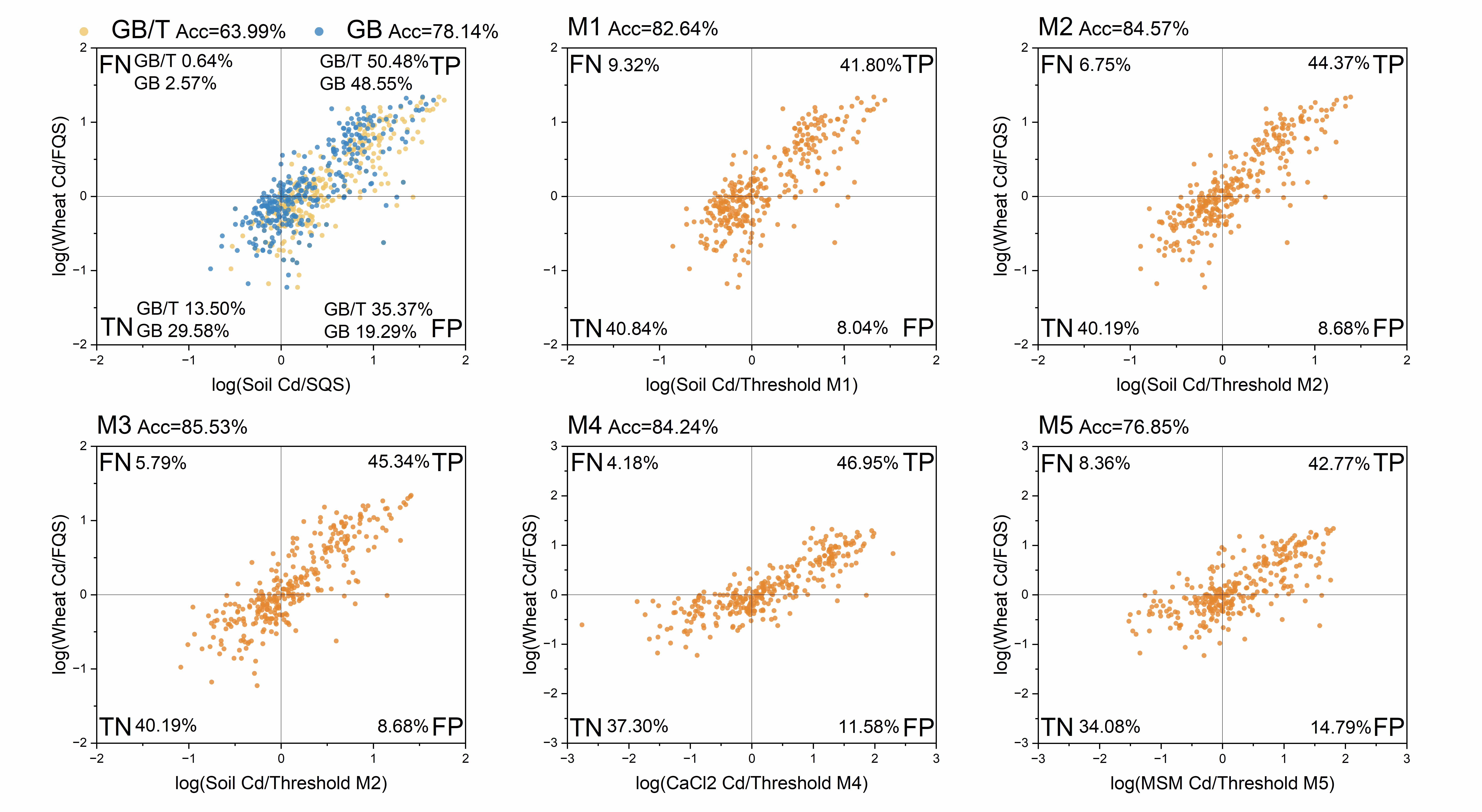


**Figure S5**. Validity assessment of current soil Cd threshold (FQS: Food Quality Standard, GB 2762-2022; SQS: Soil Quality Standard, including GB/T 41685-2022 and GB 15618-2018; TP: True Positive, FN: False Negative, FP: False Positive, TN: True Negative. ).





**Figure S6**. Calibration curves and residual analysis for eight ML models.


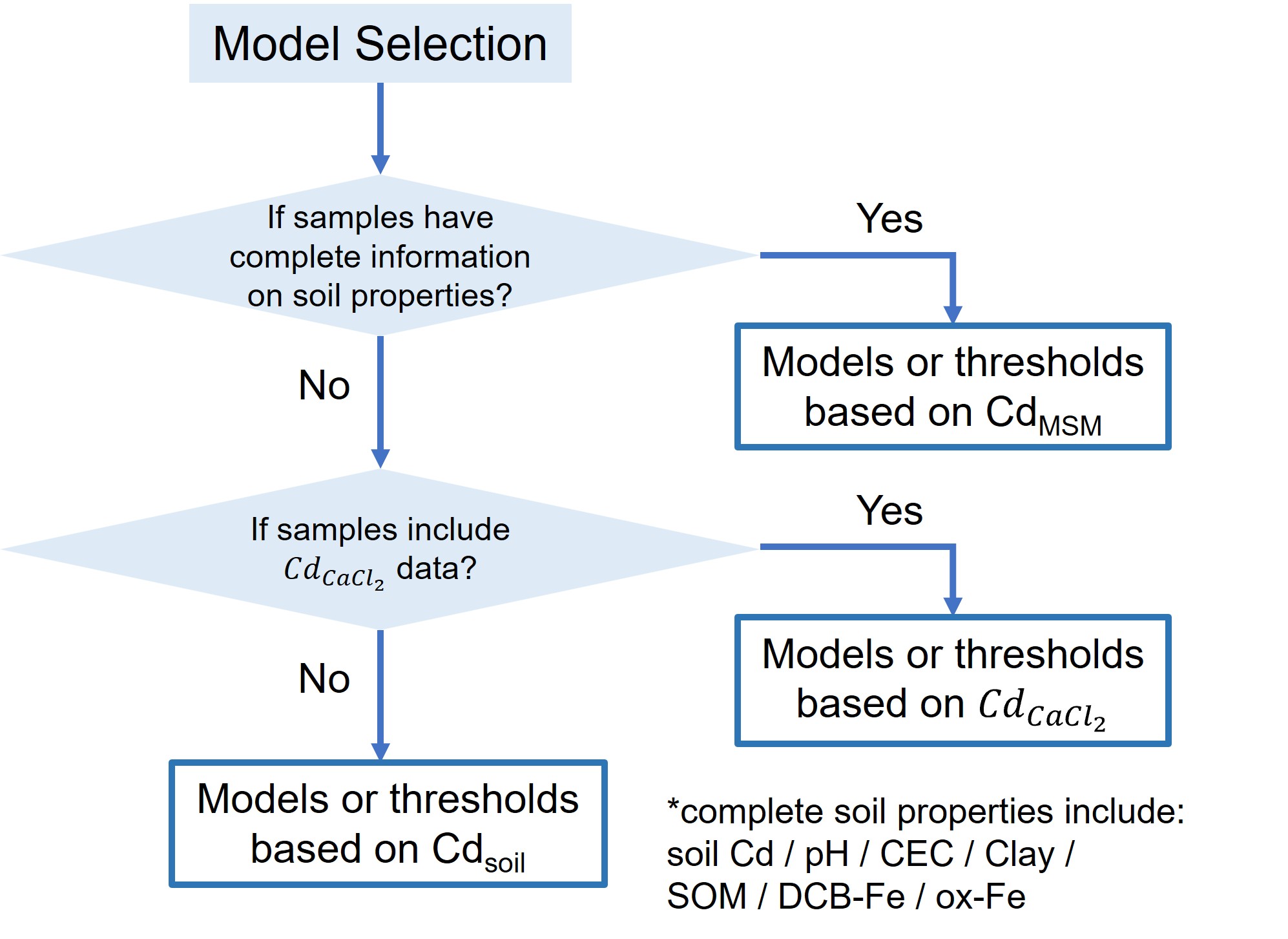


**Figure S7**. Flowchart of how to select models and thresholds in field applications.

**Table S1**. Model scenarios and their correlation coefficients with wheat Cd.

| Scenario no. | solid/liquid ratio (g L^-1^) | Major cations | R^2^ with wheat Cd |
| --- | --- | --- | --- |
| 1 | 100 | Not included | 0.336 |
| 2 | 10 | Included | 0.752 |
| 3 | 100 | Included | 0.761 |
| 4 | 1000 | Included | 0.705 |

**Table S2**. Path coefficients of PLS-PM.

| Relationships | Direct effect (λ_d_) | Indirect effect (λ_i_) | Total effect (λ_t_) |
| --- | --- | --- | --- |
| Cd_soil_ → soil pH | 0.0000 | 0.0000 | 0.0000 |
| Cd_soil_ → soil reactive surfaces | 0.0000 | 0.0000 | 0.0000 |
| Cd_soil_ → CEC | 0.0000 | 0.0000 | 0.0000 |
| Cd_soil_ → bioavailable Cd (Cd_CaCl2_) | 0.7889 | 0.0000 | 0.7889 |
| Cd_soil_ → Cd_wheat_ | 0.0000 | 0.5747 | 0.5747 |
| soil pH → soil reactive surfaces | 0.0000 | 0.0000 | 0.0000 |
| soil pH → CEC | 0.1760 | 0.0000 | 0.1760 |
| soil pH → bioavailable Cd (Cd_CaCl2_) | −0.1525 | −0.0104 | −0.1629 |
| soil pH → Cd_wheat_ | −0.0063 | −0.1187 | −0.1250 |
| soil reactive surfaces → CEC | 0.4058 | 0.0000 | 0.4058 |
| soil reactive surfaces → bioavailable Cd (Cd_CaCl2_) | 0.0029 | −0.0239 | −0.0211 |
| soil reactive surfaces → Cd_wheat_ | 0.0000 | −0.0154 | −0.0154 |
| CEC → bioavailable Cd (Cd_CaCl2_) | −0.0590 | 0.0000 | −0.0590 |
| CEC → Cd_wheat_ | 0.0000 | −0.0430 | −0.0430 |
| bioavailable Cd (Cd_CaCl2_) → Cd_wheat_ | 0.7285 | 0.0000 | 0.7285 |

**Table S3**. Models of Cd content in wheat grain and model performance (n=311).

| Equation/Algorithm | | R^2^  (training) | R^2^  (test) | RMSE  (test) | MAE  (test) |
| --- | --- | --- | --- | --- | --- |
| logCd_wheat_ = 0.930 logCd_soil_ − 0.132 pH + 0.032 | | 0.721 | 0.685 | 0.288 | 0.219 |
| logCd_wheat_ = 0.949 logCd_soil_−0.149 pH + 0.005 Clay + 0.075 | | 0.725 | 0.705 | 0.278 | 0.211 |
| logCd_wheat_ = 0.931 logCd_soil_−0.130 pH + 0.001 SOM + 0.002 | | 0.721 | 0.682 | 0.288 | 0.217 |
| logCd_wheat_ = 0.931 logCd_soil_−0.139 pH + 0.011 DCBFe − 0.002 oxFe − 0.026 | | 0.726 | 0.712 | 0.275 | 0.207 |
| logCd_wheat_ = 0.998 logCd_soil_ − 0.141 pH − 0.018 CEC + 0.007 Clay − 0.001 SOM + 0.012 DCBFe + 0.008 oxFe + 0.123 | | 0.750 | 0.765 | 0.251 | 0.198 |
| logCd_wheat_ = 0.451 logCd_CaCl2_ − 0.258 | | 0.653 | 0.701 | 0.283 | 0.231 |
| logCd_wheat_ = 0.497 logCd_MSM_ + 0.184 | | 0.570 | 0.593 | 0.327 | 0.260 |
|  | RMSE  (cross_validation) | R^2^  (training) | R^2^  (test) | RMSE  (test) | MAE  (test) |
| Ridge | 0.247 | 0.802 | 0.803 | 0.225 | 0.167 |
| DT | 0.390 | 1.000 | 0.526 | 0.350 | 0.254 |
| RF | 0.263 | 0.966 | 0.790 | 0.233 | 0.175 |
| ERT | 0.261 | 1.000 | 0.810 | 0.221 | 0.165 |
| GBDT | 0.267 | 0.968 | 0.784 | 0.236 | 0.173 |
| Xgboost | 0.285 | 1.000 | 0.725 | 0.267 | 0.204 |
| SVR | 0.417 | 0.964 | 0.431 | 0.384 | 0.299 |
| KNN | 0.401 | 1.000 | 0.431 | 0.384 | 0.299 |

Units: CEC (cmol kg^-1^), clay (%), SOM (g kg^-1^), DCB-Fe (g kg^-1^), ox-Fe (g kg^-1^), Cd_wheat_ ( mg kg^-1^), Cd_soil_ ( mg kg^-1^), Cd_CaCl2_ (mg kg^-1^) and Cd_MSM_ (mg L^-1^)

**Table S4**. Model parameters of the 8 algorithms.

|  | alpha | tol | n_estimators | min_samples _split | min_samples_leaf | max_features | learning_rate | max_depth |
| --- | --- | --- | --- | --- | --- | --- | --- | --- |
| Ridge | 1.0 | 1e-4 |  |  |  |  |  |  |
| DT |  |  |  | 2 | 1 |  |  |  |
| RF |  |  | 100 | 2 | 1 | 1.0 |  |  |
| ERT |  |  | 100 | 2 | 1 | 1.0 |  |  |
| GBRT | 0.9 | 1e-4 | 100 | 2 | 1 |  | 0.1 | 3 |
| Xgboost | 1.0 |  | 10 |  |  |  | 0.3 | 6 |

|  | tol | degree | C | gamma | epsilon | cache_size | n_neignbors | leaf_size | p |
| --- | --- | --- | --- | --- | --- | --- | --- | --- | --- |
| SVR | 1e-3 | 3 | 1.0 | 0.1 | 0.1 | 200 |  |  |  |
| KNN |  |  |  |  |  |  | 5 | 30 | 2 |

**Table S5**. The effect of the number of trees in ERT on model predictions.

| Number of trees | R^2^  (training) | R^2^  (test) | RMSE  (test) | MAE  (test) |
| --- | --- | --- | --- | --- |
| 50 | 1.000 | 0.785 | 0.235 | 0.171 |
| 60 | 1.000 | 0.787 | 0.233 | 0.171 |
| 70 | 1.000 | 0.793 | 0.230 | 0.169 |
| 80 | 1.000 | 0.796 | 0.229 | 0.169 |
| 90 | 1.000 | 0.803 | 0.225 | 0.167 |
| 100 | 1.000 | 0.810 | 0.221 | 0.165 |
| 110 | 1.000 | 0.810 | 0.220 | 0.165 |
| 120 | 1.000 | 0.811 | 0.220 | 0.166 |

**Table S6**. Packages used in scikit-learn.

|  |  | Underlying package |
| --- | --- | --- |
| Model | Ridge | Ridge of sklearn.linear_model |
|  | DT | DecisionTreeRegressor of sklearn.tree |
|  | RF | RandomForestRegressor of sklearn.ensemble |
|  | ERT | ExtraTreesRegressor of sklearn.ensemble |
|  | GBRT | GradientBoostingRegressor of sklearn.ensemble |
|  | Xgboost | XGBRegressor of xgboost |
|  | SVR | SVR of sklearn.svm |
|  | KNN | KNeighborsRegressor of sklearn.neighbors |
| Performance evaluator | R^2^ | r2_score of sklearn.metrics |
|  | RMSE | mean_squared_error of sklearn.metrics |
|  | MAE | mean_absolute_error of sklearn.metrics |

**Table S7**. Performance metrics of 10 independent random splits.

| Model | R^2^ | RMSE | MAE |
| --- | --- | --- | --- |
|  | mean ± standard deviation | | |
| M1 | 0.640±0.058 | 0.314±0.027 | 0.248±0.021 |
| M2 | 0.717±0.047 | 0.278±0.021 | 0.208±0.016 |
| M3 | 0.722±0.049 | 0.275±0.054 | 0.227±0.036 |
| M4 | 0.696±0.054 | 0.288±0.023 | 0.222±0.017 |
| M5 | 0.631±0.044 | 0.318±0.018 | 0.253±0.018 |
| M6(ERT) | 0.792±0.020 | 0.239±0.008 | 0.179±0.009 |
| Ridge | 0.754±0.045 | 0.259±0.020 | 0.197±0.016 |
| DT | 0.520±0.083 | 0.361±0.022 | 0.259±0.017 |
| RF | 0.770±0.033 | 0.250±0.013 | 0.187±0.010 |
| GBDT | 0.760±0.030 | 0.257±0.014 | 0.193±0.012 |
| Xgboost | 0.727±0.035 | 0.273±0.017 | 0.205±0.013 |
| SVR | 0.363±0.076 | 0.418±0.025 | 0.322±0.022 |
| KNN | 0.376±0.072 | 0.414±0.016 | 0.312±0.013 |

**Table S8**. Models of Cd content in wheat grain and 95% confidence intervals for coefficients.

| Model | Equation/Algorithm |
| --- | --- |
| M1 | logCd_wheat_ = 0.906 [0.821, 0.991] logCd_soil_ − 0.718 [-0.761, -0.674] |
| M2 | logCd_wheat_ = 0.955 [0.879, 1.031] logCd_soil_ − 0.118 [-0.151, -0.086] pH − 0.010 [-0.017, -0.003] CEC + 0.114 [-0.075, 0.303] |
| M3 | Cd_wheat_ = BCF × Cd_soil_  BCF = 0.014 [-0.001, 0.028] pH^2^ − 0.236 [-0.407, -0.052] pH + 1.113 [0.566, 1.632] |
| M4 | logCd_wheat_ = 0.509 [0.465, 0.554] logCd_CaCl2_ + 0.104 [0.066, 0.141] pH − 0.768 [-0.963, -0.573] |
| M5 | logCd_wheat_ = 0.572 [0.512, 0.632] logCd_MSM_ + 0.106 [0.063, 0.149] pH − 0.257 [-0.472, -0.042] |
| M5* | logCd_wheat_ = 0.690 [0.623, 0.757] logCd_MSM_ + 0.020 [-0.029,0.068] pH + 0.385 [0.123, 0.647] |

The 95% confidence intervals for the model coefficients are given in [].

**Table S9**. The data of the soil and wheat samples (n=311).

|  | **Data sources** | **logCd_soil_** | **Soil pH** | **logCd_CaCl2_** | **Clay** | **CEC** | **SOM** | **DCB-Fe** | **ox-Fe** | **logCd_wheat_** | **BCF** | **logCd_MSM_** |
| --- | --- | --- | --- | --- | --- | --- | --- | --- | --- | --- | --- | --- |
|  |  | mg kg^-1^ |  | mg kg^-1^ | % | cmol kg^-1^ | g kg^-1^ | g kg^-1^ | g kg^-1^ | mg kg^-1^ |  | mg kg^-1^ |
| 1 | Dataset 1 | 0.467 | 7.75 | 0.258 | 16.910 | 18.452 | 14.000 | 7.037 | 0.762 | -0.167 | 0.232 | -1.442 |
| 2 | Dataset 1 | -0.137 | 5.35 | -1.008 | 18.520 | 9.150 | 28.500 | 13.488 | 0.917 | -1.051 | 0.122 | -1.991 |
| 3 | Dataset 1 | 0.167 | 6.05 | -1.112 | 21.520 | 18.450 | 46.580 | 8.800 | 4.217 | -0.790 | 0.110 | -2.223 |
| 4 | Dataset 1 | 0.100 | 8.55 | -1.187 | 27.000 | 3.255 | 30.100 | 8.924 | 0.928 | -0.666 | 0.171 | -3.068 |
| 5 | Dataset 1 | 0.297 | 8.18 | -0.816 | 17.200 | 5.520 | 17.310 | 7.037 | 0.762 | -0.301 | 0.253 | -2.140 |
| 6 | Dataset 1 | 0.297 | 8.18 | -0.839 | 17.200 | 5.520 | 17.310 | 7.037 | 0.762 | -0.237 | 0.293 | -2.140 |
| 7 | Dataset 1 | 0.344 | 8.23 | -0.797 | 18.500 | 12.920 | 22.850 | 7.037 | 0.762 | -0.370 | 0.193 | -2.236 |
| 8 | Dataset 1 | 0.344 | 8.23 | -0.840 | 18.500 | 12.920 | 22.850 | 7.037 | 0.762 | -0.242 | 0.259 | -2.236 |
| 9 | Dataset 1 | 0.623 | 8.45 | -0.619 | 14.300 | 6.779 | 19.120 | 8.924 | 0.928 | -0.176 | 0.159 | -1.991 |
| 10 | Dataset 1 | 0.623 | 8.45 | -0.525 | 14.300 | 6.779 | 19.120 | 8.924 | 0.928 | 0.041 | 0.262 | -1.991 |
| 11 | Dataset 1 | 0.408 | 8.14 | -0.309 | 15.590 | 3.255 | 28.400 | 8.924 | 0.928 | -0.013 | 0.379 | -2.135 |
| 12 | Dataset 1 | 0.629 | 7.80 | -1.460 | 21.040 | 20.000 | 26.400 | 8.924 | 0.928 | -0.618 | 0.057 | -1.544 |
| 13 | Dataset 1 | -0.066 | 6.46 | -1.824 | 22.080 | 18.660 | 24.770 | 9.490 | 2.300 | -0.473 | 0.391 | -2.701 |
| 14 | Dataset 1 | -0.433 | 8.20 | -3.068 | 42.700 | 17.770 | 19.760 | 14.700 | 8.367 | -1.134 | 0.199 | -3.670 |
| 15 | Dataset 1 | -0.380 | 7.59 | -2.892 | 36.000 | 20.980 | 54.470 | 14.700 | 8.367 | -1.330 | 0.112 | -3.889 |
| 16 | Dataset 1 | -0.467 | 7.78 | -3.068 | 27.700 | 14.570 | 40.160 | 14.700 | 8.367 | -1.397 | 0.118 | -3.936 |
| 17 | Dataset 1 | -0.552 | 8.26 | -3.068 | 28.700 | 13.130 | 18.550 | 14.700 | 8.367 | -1.427 | 0.133 | -3.836 |
| 18 | Dataset 1 | -0.564 | 7.09 | -2.716 | 33.200 | 21.810 | 33.830 | 24.200 | 7.700 | -1.427 | 0.137 | -3.493 |
| 19 | Dataset 1 | -0.524 | 6.17 | -1.636 | 24.300 | 17.220 | 30.070 | 24.200 | 7.700 | -0.565 | 0.911 | -3.152 |
| 20 | Dataset 1 | -0.495 | 5.88 | -1.488 | 33.200 | 20.700 | 47.140 | 8.800 | 4.217 | -0.841 | 0.451 | -3.202 |
| 21 | Dataset 1 | -0.693 | 6.99 | -2.670 | 39.800 | 20.580 | 20.790 | 24.200 | 7.700 | -1.619 | 0.119 | -3.288 |
| 22 | Dataset 1 | -0.804 | 6.85 | -2.439 | 34.800 | 18.340 | 19.850 | 15.000 | 6.083 | -1.573 | 0.170 | -3.327 |
| 23 | Dataset 1 | -0.516 | 5.98 | -1.670 | 35.200 | 23.400 | 38.650 | 16.500 | 7.267 | -0.945 | 0.372 | -3.177 |
| 24 | Dataset 1 | -0.853 | 5.98 | -1.989 | 32.600 | 21.710 | 28.300 | 15.000 | 6.083 | -1.343 | 0.324 | -3.506 |
| 25 | Dataset 1 | -0.577 | 8.02 | -3.193 | 35.500 | 24.530 | 40.960 | 12.300 | 10.367 | -1.356 | 0.166 | -4.287 |
| 26 | Dataset 1 | -0.783 | 5.61 | -1.576 | 24.100 | 19.740 | 37.480 | 4.300 | 2.840 | -1.221 | 0.365 | -3.305 |
| 27 | Dataset 1 | -0.426 | 8.15 | -2.971 | 31.600 | 17.720 | 40.170 | 14.700 | 8.367 | -1.011 | 0.260 | -4.072 |
| 28 | Dataset 1 | -0.937 | 5.98 | -1.740 | 30.600 | 15.730 | 20.700 | 11.300 | 4.783 | -1.343 | 0.393 | -3.421 |
| 29 | Dataset 1 | -0.372 | 8.28 | -3.068 | 31.200 | 14.420 | 29.860 | 14.700 | 8.367 | -1.126 | 0.176 | -3.845 |
| 30 | Dataset 1 | -0.477 | 8.04 | -3.068 | 27.900 | 15.230 | 32.420 | 14.700 | 8.367 | -1.241 | 0.172 | -3.939 |
| 31 | Dataset 1 | -0.859 | 7.62 | -3.068 | 13.600 | 11.560 | 27.120 | 14.700 | 8.367 | -1.532 | 0.213 | -4.235 |
| 32 | Dataset 1 | -0.706 | 6.67 | -2.369 | 23.200 | 15.040 | 28.470 | 6.400 | 4.367 | -1.459 | 0.176 | -3.263 |
| 33 | Dataset 1 | -0.359 | 8.31 | -3.068 | 19.200 | 11.490 | 26.370 | 16.300 | 9.083 | -1.330 | 0.107 | -3.750 |
| 34 | Dataset 1 | -0.643 | 7.81 | -2.892 | 18.200 | 11.240 | 24.220 | 12.100 | 7.667 | -1.383 | 0.182 | -3.886 |
| 35 | Dataset 1 | -0.521 | 8.31 | -3.670 | 44.700 | 20.860 | 21.930 | 18.300 | 4.567 | -1.221 | 0.200 | -3.914 |
| 36 | Dataset 1 | -0.380 | 7.86 | -3.670 | 56.700 | 28.370 | 42.370 | 9.700 | 8.550 | -1.126 | 0.180 | -3.876 |
| 37 | Dataset 1 | -0.865 | 4.95 | -1.617 | 21.800 | 15.640 | 30.920 | 13.000 | 4.117 | -1.619 | 0.176 | -2.917 |
| 38 | Dataset 1 | -0.415 | 8.02 | -2.892 | 55.300 | 28.250 | 49.710 | 13.900 | 8.667 | -1.397 | 0.104 | -4.148 |
| 39 | Dataset 1 | -0.487 | 8.28 | -3.193 | 54.700 | 27.840 | 39.000 | 13.900 | 8.667 | -1.795 | 0.049 | -4.241 |
| 40 | Dataset 1 | -0.580 | 8.23 | -3.670 | 49.500 | 27.520 | 29.610 | 17.300 | 5.650 | -2.175 | 0.025 | -4.168 |
| 41 | Dataset 1 | -0.419 | 8.49 | -3.193 | 40.400 | 22.140 | 30.740 | 8.500 | 6.550 | -1.728 | 0.049 | -4.063 |
| 42 | Dataset 1 | -0.640 | 7.25 | -2.716 | 26.600 | 17.930 | 23.870 | 12.000 | 7.600 | -1.670 | 0.093 | -3.492 |
| 43 | Dataset 1 | -0.697 | 8.12 | -3.193 | 24.600 | 14.880 | 28.140 | 9.100 | 7.900 | -1.728 | 0.093 | -4.266 |
| 44 | Dataset 1 | -0.292 | 6.01 | -1.237 | 28.000 | 19.600 | 33.550 | 13.700 | 1.010 | -0.682 | 0.408 | -2.987 |
| 45 | Dataset 1 | -0.292 | 6.01 | -1.237 | 28.000 | 19.600 | 33.550 | 13.700 | 1.010 | -0.798 | 0.313 | -2.987 |
| 46 | Dataset 1 | -0.548 | 6.80 | -1.131 | 8.560 | 11.400 | 29.500 | 10.100 | 0.980 | -0.854 | 0.495 | -3.056 |
| 47 | Dataset 2 | -1.020 | 6.88 | -3.120 | 14.095 | 7.574 | 8.280 | 20.242 | 4.068 | -1.166 | 0.716 | -3.030 |
| 48 | Dataset 2 | -0.987 | 7.74 | -3.358 | 12.146 | 10.202 | 0.605 | 15.043 | 5.548 | -1.976 | 0.103 | -2.777 |
| 49 | Dataset 2 | -0.471 | 5.94 | -2.457 | 11.959 | 12.769 | 31.748 | 9.289 | 4.207 | -1.065 | 0.255 | -2.790 |
| 50 | Dataset 2 | -0.404 | 6.05 | -2.168 | 12.027 | 12.939 | 21.949 | 11.299 | 4.114 | -1.272 | 0.135 | -2.547 |
| 51 | Dataset 2 | -0.157 | 5.50 | -1.962 | 12.855 | 13.780 | 60.886 | 10.162 | 5.944 | -0.880 | 0.189 | -2.465 |
| 52 | Dataset 2 | -0.216 | 4.91 | -1.162 | 13.833 | 11.917 | 38.649 | 12.081 | 6.218 | -0.831 | 0.243 | -2.003 |
| 53 | Dataset 2 | -1.168 | 6.42 | -3.083 | 3.723 | 7.804 | 5.183 | 13.563 | 2.347 | -1.672 | 0.313 | -3.192 |
| 54 | Dataset 2 | -0.368 | 4.64 | -1.144 | 13.955 | 15.395 | 50.029 | 11.344 | 8.769 | -0.880 | 0.308 | -2.201 |
| 55 | Dataset 2 | -0.495 | 4.34 | -1.148 | 11.695 | 12.461 | 28.750 | 12.509 | 6.745 | -0.924 | 0.372 | -2.018 |
| 56 | Dataset 2 | -0.318 | 5.92 | -2.524 | 13.653 | 17.441 | 44.801 | 11.352 | 5.892 | -1.032 | 0.193 | -2.631 |
| 57 | Dataset 2 | -0.423 | 4.39 | -1.147 | 12.810 | 14.161 | 44.029 | 9.433 | 7.536 | -0.818 | 0.402 | -2.084 |
| 58 | Dataset 2 | 0.044 | 5.37 | -1.263 | 12.547 | 18.217 | 39.102 | 9.484 | 7.271 | -0.939 | 0.104 | -1.728 |
| 59 | Dataset 2 | -0.408 | 6.78 | -3.035 | 12.415 | 14.854 | 58.989 | 9.210 | 6.716 | -1.075 | 0.215 | -3.148 |
| 60 | Dataset 2 | -0.502 | 4.71 | -1.462 | 14.019 | 11.251 | 45.038 | 11.921 | 5.515 | -0.872 | 0.427 | -2.372 |
| 61 | Dataset 2 | -0.217 | 4.64 | -1.225 | 13.009 | 15.164 | 35.805 | 12.426 | 8.240 | -0.862 | 0.227 | -1.772 |
| 62 | Dataset 2 | -0.321 | 4.69 | -1.231 | 12.814 | 14.587 | 33.242 | 11.392 | 8.262 | -0.766 | 0.358 | -1.903 |
| 63 | Dataset 2 | -0.249 | 4.75 | -1.266 | 15.070 | 14.641 | 41.441 | 11.959 | 8.674 | -0.768 | 0.302 | -1.936 |
| 64 | Dataset 2 | -0.290 | 4.48 | -1.109 | 12.934 | 14.970 | 47.082 | 11.000 | 7.141 | -0.756 | 0.342 | -1.932 |
| 65 | Dataset 2 | -0.359 | 4.67 | -1.306 | 11.808 | 13.297 | 58.818 | 8.321 | 6.863 | -1.003 | 0.227 | -2.268 |
| 66 | Dataset 2 | -0.443 | 4.75 | -1.363 | 14.016 | 14.486 | 30.047 | 11.872 | 7.758 | -0.925 | 0.330 | -2.031 |
| 67 | Dataset 2 | -0.250 | 5.53 | -2.365 | 13.361 | 15.274 | 47.418 | 10.326 | 7.725 | -0.942 | 0.203 | -2.337 |
| 68 | Dataset 2 | -0.416 | 5.34 | -1.897 | 13.255 | 11.673 | 35.419 | 8.043 | 5.918 | -1.394 | 0.105 | -2.363 |
| 69 | Dataset 2 | -0.764 | 5.91 | -2.906 | 8.952 | 9.848 | 28.895 | 9.298 | 2.968 | -1.357 | 0.256 | -3.124 |
| 70 | Dataset 2 | 0.162 | 6.87 | -2.380 | 12.835 | 8.494 | 49.478 | 13.602 | 8.904 | -1.002 | 0.068 | -2.076 |
| 71 | Dataset 2 | -0.389 | 4.72 | -1.324 | 11.018 | 12.670 | 50.934 | 8.186 | 6.545 | -1.017 | 0.235 | -2.264 |
| 72 | Dataset 2 | -0.543 | 4.20 | -1.387 | 12.310 | 12.128 | 35.835 | 8.085 | 7.332 | -1.276 | 0.185 | -2.087 |
| 73 | Dataset 2 | 0.158 | 4.36 | -1.439 | 12.745 | 9.852 | 31.878 | 8.192 | 7.583 | -1.017 | 0.067 | -1.225 |
| 74 | Dataset 2 | 0.152 | 4.14 | -0.449 | 9.096 | 7.272 | 56.188 | 7.182 | 5.656 | -0.269 | 0.379 | -1.355 |
| 75 | Dataset 2 | -0.541 | 4.58 | -1.480 | 8.860 | 15.480 | 46.481 | 9.223 | 7.876 | -1.278 | 0.183 | -2.333 |
| 76 | Dataset 2 | -0.616 | 5.05 | -1.670 | 8.090 | 10.467 | 60.880 | 6.059 | 4.785 | -1.056 | 0.363 | -2.857 |
| 77 | Dataset 2 | -0.443 | 4.50 | -1.300 | 9.533 | 11.688 | 26.670 | 8.146 | 5.678 | -0.929 | 0.327 | -1.932 |
| 78 | Dataset 2 | -0.387 | 4.91 | -1.726 | 13.203 | 5.059 | 44.216 | 9.861 | 7.165 | -1.253 | 0.136 | -2.201 |
| 79 | Dataset 2 | -0.697 | 5.16 | -2.104 | 13.248 | 13.736 | 32.439 | 10.066 | 6.404 | -1.394 | 0.201 | -2.561 |
| 80 | Dataset 2 | -0.623 | 5.04 | -1.843 | 12.833 | 13.040 | 29.522 | 10.181 | 6.833 | -1.207 | 0.260 | -2.382 |
| 81 | Dataset 2 | -0.510 | 4.66 | -1.705 | 13.624 | 14.553 | 41.702 | 11.087 | 6.527 | -0.972 | 0.345 | -2.211 |
| 82 | Dataset 2 | -0.352 | 6.83 | -3.516 | 6.495 | 10.331 | 14.096 | 7.950 | 4.887 | -1.893 | 0.029 | -2.202 |
| 83 | Dataset 2 | -0.641 | 4.59 | -1.607 | 9.607 | 10.800 | 14.289 | 9.166 | 5.029 | -1.199 | 0.277 | -2.005 |
| 84 | Dataset 2 | -0.493 | 4.68 | -1.807 | 5.903 | 9.606 | 23.798 | 6.199 | 5.101 | -1.268 | 0.168 | -2.032 |
| 85 | Dataset 2 | -0.373 | 5.18 | -2.336 | 6.648 | 11.269 | 31.365 | 5.942 | 4.864 | -1.270 | 0.127 | -2.164 |
| 86 | Dataset 2 | -0.362 | 4.98 | -1.650 | 6.330 | 9.249 | 20.421 | 7.384 | 4.673 | -1.271 | 0.123 | -1.870 |
| 87 | Dataset 2 | -0.409 | 4.90 | -1.539 | 5.241 | 8.560 | 36.889 | 6.176 | 4.536 | -1.335 | 0.118 | -2.197 |
| 88 | Dataset 2 | -0.557 | 4.05 | -1.221 | 9.079 | 12.259 | 41.180 | 6.993 | 6.323 | -1.008 | 0.354 | -2.089 |
| 89 | Dataset 2 | -0.567 | 4.76 | -1.460 | 8.918 | 11.649 | 38.429 | 6.872 | 5.663 | -1.323 | 0.176 | -2.337 |
| 90 | Dataset 2 | -0.580 | 4.28 | -1.242 | 6.149 | 8.736 | 28.610 | 6.581 | 4.752 | -1.178 | 0.252 | -2.078 |
| 91 | Dataset 2 | -0.700 | 4.49 | -1.386 | 5.826 | 7.356 | 28.245 | 7.050 | 4.269 | -1.086 | 0.412 | -2.305 |
| 92 | Dataset 2 | -0.657 | 4.24 | -1.366 | 5.659 | 7.486 | 29.817 | 6.383 | 3.955 | -1.231 | 0.267 | -2.194 |
| 93 | Dataset 2 | -0.647 | 3.97 | -1.229 | 7.959 | 8.021 | 38.052 | 7.616 | 4.636 | -1.102 | 0.351 | -2.159 |
| 94 | Dataset 2 | 0.208 | 4.09 | -0.340 | 10.391 | 11.427 | 48.261 | 8.583 | 4.647 | -0.370 | 0.264 | -1.220 |
| 95 | Dataset 2 | 0.189 | 4.51 | -0.600 | 6.250 | 11.714 | 23.120 | 9.109 | 4.349 | -0.291 | 0.331 | -1.097 |
| 96 | Dataset 2 | 0.833 | 4.09 | 0.010 | 9.703 | 13.331 | 29.864 | 8.446 | 6.743 | 0.325 | 0.310 | -0.364 |
| 97 | Dataset 2 | 0.053 | 4.07 | -0.550 | 7.741 | 9.479 | 63.510 | 7.094 | 5.063 | -0.396 | 0.355 | -1.521 |
| 98 | Dataset 2 | -0.269 | 5.36 | -2.150 | 9.946 | 12.539 | 42.459 | 9.044 | 5.808 | -0.987 | 0.192 | -2.236 |
| 99 | Dataset 2 | -0.262 | 4.87 | -1.112 | 8.364 | 8.903 | 33.144 | 11.317 | 2.200 | -0.999 | 0.183 | -1.932 |
| 100 | Dataset 2 | 0.372 | 4.54 | -0.337 | 10.120 | 10.294 | 40.216 | 7.426 | 4.964 | 0.048 | 0.474 | -1.082 |
| 101 | Dataset 2 | 0.373 | 4.43 | 0.025 | 8.497 | 11.997 | 28.260 | 11.004 | 5.392 | 0.198 | 0.669 | -0.947 |
| 102 | Dataset 2 | 0.774 | 4.03 | 0.167 | 10.205 | 8.152 | 48.260 | 7.903 | 5.408 | 0.215 | 0.276 | -0.521 |
| 103 | Dataset 2 | 0.542 | 4.51 | -0.219 | 11.322 | 10.712 | 45.533 | 7.101 | 5.183 | 0.017 | 0.299 | -0.932 |
| 104 | Dataset 2 | 0.718 | 4.79 | -0.317 | 10.904 | 13.852 | 55.909 | 8.088 | 5.654 | 0.077 | 0.229 | -0.851 |
| 105 | Dataset 2 | 0.827 | 4.73 | -0.165 | 14.243 | 15.345 | 45.753 | 12.277 | 6.631 | -0.269 | 0.080 | -0.613 |
| 106 | Dataset 2 | 0.239 | 4.77 | -0.573 | 13.002 | 16.203 | 48.753 | 10.342 | 6.545 | 0.049 | 0.646 | -1.365 |
| 107 | Dataset 2 | -0.302 | 4.53 | -1.212 | 15.670 | 13.905 | 45.665 | 12.155 | 8.006 | -1.080 | 0.167 | -1.967 |
| 108 | Dataset 2 | -0.328 | 4.77 | -1.103 | 5.905 | 16.114 | 48.703 | 10.548 | 7.810 | -0.988 | 0.219 | -2.121 |
| 109 | Dataset 2 | -0.722 | 4.24 | -1.372 | 6.767 | 7.126 | 31.080 | 6.548 | 4.895 | -1.294 | 0.268 | -2.270 |
| 110 | Dataset 2 | -0.516 | 4.18 | -1.185 | 14.129 | 14.722 | 41.148 | 11.014 | 6.565 | -1.054 | 0.290 | -2.078 |
| 111 | Dataset 2 | -0.437 | 4.55 | -1.441 | 9.382 | 11.180 | 33.545 | 8.641 | 6.289 | -1.373 | 0.116 | -2.051 |
| 112 | Dataset 2 | -0.606 | 4.16 | -1.327 | 11.621 | 12.349 | 32.029 | 8.494 | 6.948 | -1.165 | 0.276 | -1.947 |
| 113 | Dataset 2 | -0.443 | 6.44 | -2.845 | 9.124 | 13.100 | 34.884 | 7.699 | 5.678 | -2.060 | 0.024 | -3.099 |
| 114 | Dataset 2 | -0.460 | 6.21 | -2.613 | 8.318 | 11.534 | 25.824 | 6.824 | 4.524 | -2.224 | 0.017 | -2.750 |
| 115 | Dataset 2 | -0.552 | 4.63 | -1.972 | 8.710 | 10.694 | 43.823 | 7.478 | 4.392 | -1.246 | 0.202 | -2.376 |
| 116 | Dataset 2 | -0.625 | 6.04 | -3.186 | 9.380 | 9.581 | 18.294 | 8.744 | 3.649 | -1.634 | 0.098 | -2.733 |
| 117 | Dataset 2 | -0.579 | 4.39 | -1.305 | 6.241 | 10.263 | 27.354 | 6.851 | 5.263 | -1.328 | 0.178 | -2.109 |
| 118 | Dataset 2 | -0.570 | 4.38 | -1.451 | 5.306 | 9.867 | 27.484 | 6.777 | 5.175 | -1.086 | 0.305 | -2.091 |
| 119 | Dataset 2 | -0.600 | 4.17 | -1.505 | 6.758 | 10.477 | 41.572 | 5.344 | 3.679 | -0.772 | 0.674 | -2.246 |
| 120 | Dataset 2 | -0.647 | 4.23 | -1.422 | 11.738 | 12.792 | 34.546 | 9.024 | 7.401 | -1.087 | 0.363 | -2.202 |
| 121 | Dataset 2 | -0.438 | 4.16 | -1.147 | 12.481 | 14.558 | 42.109 | 9.879 | 6.977 | -1.010 | 0.268 | -1.979 |
| 122 | Dataset 2 | -0.351 | 4.92 | -1.346 | 7.655 | 13.211 | 61.758 | 9.397 | 5.985 | -0.853 | 0.315 | -2.378 |
| 123 | Dataset 2 | -0.423 | 4.11 | -1.104 | 11.533 | 8.668 | 28.461 | 7.149 | 5.512 | -0.905 | 0.330 | -1.851 |
| 124 | Dataset 2 | 0.045 | 4.56 | -0.645 | 7.841 | 5.852 | 33.144 | 7.570 | 4.614 | -0.359 | 0.395 | -1.453 |
| 125 | Dataset 2 | -0.317 | 6.46 | -3.046 | 6.239 | 7.279 | 12.762 | 9.126 | 5.635 | -1.363 | 0.090 | -2.229 |
| 126 | Dataset 2 | -0.337 | 4.55 | -1.060 | 11.796 | 13.475 | 39.077 | 14.681 | 7.039 | -0.827 | 0.324 | -1.991 |
| 127 | Dataset 2 | -0.281 | 4.55 | -1.012 | 12.577 | 13.714 | 45.079 | 12.732 | 8.051 | -0.785 | 0.313 | -1.967 |
| 128 | Dataset 2 | -0.241 | 5.34 | -1.351 | 13.489 | 15.160 | 44.649 | 11.708 | 8.306 | -0.879 | 0.230 | -2.175 |
| 129 | Dataset 2 | -0.441 | 5.00 | -1.497 | 13.006 | 6.579 | 43.496 | 10.565 | 6.301 | -1.042 | 0.250 | -2.312 |
| 130 | Dataset 2 | -0.449 | 6.18 | -2.586 | 9.469 | 9.234 | 20.570 | 13.907 | 2.555 | -1.056 | 0.247 | -2.627 |
| 131 | Dataset 2 | -0.473 | 4.58 | -0.484 | 8.940 | 7.983 | 30.234 | 13.779 | 2.729 | -0.995 | 0.300 | -2.116 |
| 132 | Dataset 2 | -0.446 | 5.57 | -1.905 | 14.061 | 9.041 | 31.867 | 13.827 | 2.364 | -1.043 | 0.253 | -2.529 |
| 133 | Dataset 2 | -0.485 | 5.02 | -1.514 | 8.729 | 6.698 | 14.898 | 13.769 | 4.508 | -1.201 | 0.192 | -2.068 |
| 134 | Dataset 2 | -0.175 | 5.64 | -1.611 | 11.707 | 11.962 | 20.125 | 7.499 | 4.026 | -1.055 | 0.132 | -1.924 |
| 135 | Dataset 2 | -0.462 | 7.25 | -2.809 | 12.761 | 13.392 | 2.980 | 10.726 | 9.314 | -1.235 | 0.169 | -2.192 |
| 136 | Dataset 2 | -0.562 | 4.28 | -1.303 | 10.709 | 10.153 | 24.718 | 8.966 | 5.384 | -1.180 | 0.241 | -2.037 |
| 137 | Dataset 2 | -0.508 | 5.21 | -1.869 | 11.656 | 12.410 | 32.191 | 10.350 | 8.338 | -1.375 | 0.136 | -2.424 |
| 138 | Dataset 2 | -0.708 | 4.15 | -1.231 | 12.732 | 9.848 | 23.487 | 19.461 | 4.426 | -1.127 | 0.381 | -2.164 |
| 139 | Dataset 2 | -0.807 | 7.02 | -3.268 | 13.541 | 10.071 | 5.351 | 19.437 | 3.312 | -1.699 | 0.128 | -2.506 |
| 140 | Dataset 2 | -0.646 | 4.77 | -1.949 | 11.852 | 12.910 | 36.046 | 8.808 | 6.987 | -1.165 | 0.303 | -2.439 |
| 141 | Dataset 2 | -0.561 | 5.33 | -2.321 | 12.255 | 13.733 | 17.500 | 8.181 | 7.188 | -1.136 | 0.266 | -2.200 |
| 142 | Dataset 2 | -0.593 | 6.22 | -3.461 | 10.598 | 13.720 | 35.699 | 7.811 | 5.953 | -1.401 | 0.156 | -3.287 |
| 143 | Dataset 2 | -0.651 | 4.33 | -1.634 | 14.219 | 13.985 | 33.496 | 10.859 | 7.975 | -1.062 | 0.388 | -2.208 |
| 144 | Dataset 2 | -0.725 | 4.38 | -1.611 | 15.044 | 12.464 | 1.165 | 10.405 | 6.738 | -1.126 | 0.397 | -1.772 |
| 145 | Dataset 2 | -0.612 | 4.79 | -2.130 | 14.824 | 14.529 | 44.548 | 10.684 | 7.070 | -1.147 | 0.292 | -2.406 |
| 146 | Dataset 2 | -0.605 | 6.24 | -4.493 | 12.124 | 13.425 | 30.650 | 9.995 | 6.324 | -1.607 | 0.099 | -3.133 |
| 147 | Dataset 2 | -0.207 | 4.28 | -0.903 | 10.859 | 11.041 | 33.753 | 6.951 | 5.378 | -0.656 | 0.356 | -1.660 |
| 148 | Dataset 2 | -0.656 | 4.97 | -1.745 | 13.872 | 14.523 | 32.996 | 11.053 | 6.910 | -1.194 | 0.290 | -2.424 |
| 149 | Dataset 2 | -0.683 | 4.35 | -1.486 | 15.121 | 14.118 | 36.835 | 9.007 | 6.509 | -1.132 | 0.356 | -2.284 |
| 150 | Dataset 2 | -0.736 | 4.63 | -1.531 | 8.275 | 9.044 | 26.183 | 6.627 | 4.519 | -1.261 | 0.299 | -2.375 |
| 151 | Dataset 2 | -0.756 | 4.44 | -1.366 | 6.067 | 9.116 | 41.333 | 7.013 | 5.225 | -1.227 | 0.338 | -2.542 |
| 152 | Dataset 2 | -0.721 | 4.33 | -1.529 | 12.612 | 13.672 | 54.554 | 9.459 | 7.482 | -1.043 | 0.477 | -2.553 |
| 153 | Dataset 2 | -0.764 | 4.51 | -1.665 | 13.048 | 13.702 | 35.359 | 9.253 | 7.508 | -1.065 | 0.500 | -2.462 |
| 154 | Dataset 2 | -0.811 | 4.71 | -1.656 | 9.243 | 9.199 | 38.300 | 7.695 | 5.520 | -1.167 | 0.440 | -2.697 |
| 155 | Dataset 2 | 0.158 | 4.39 | -0.405 | 8.433 | 10.216 | 20.458 | 7.253 | 5.153 | -0.085 | 0.572 | -1.105 |
| 156 | Dataset 2 | 0.379 | 4.31 | -0.376 | 9.863 | 10.159 | 33.466 | 6.330 | 4.876 | 0.147 | 0.586 | -0.959 |
| 157 | Dataset 2 | -0.382 | 4.50 | -1.081 | 8.129 | 11.463 | 48.057 | 7.765 | 5.332 | -0.407 | 0.943 | -2.126 |
| 158 | Dataset 2 | -0.396 | 4.30 | -1.026 | 10.287 | 11.529 | 42.693 | 6.620 | 5.132 | -1.102 | 0.197 | -2.003 |
| 159 | Dataset 2 | -0.335 | 5.04 | -1.315 | 10.099 | 11.830 | 50.746 | 7.751 | 4.981 | -0.980 | 0.226 | -2.296 |
| 160 | Dataset 2 | -0.587 | 4.16 | -1.100 | 11.593 | 10.440 | 50.116 | 8.651 | 5.970 | -1.161 | 0.267 | -2.240 |
| 161 | Dataset 2 | -0.188 | 4.95 | -0.904 | 8.924 | 11.974 | 34.787 | 7.962 | 4.939 | -0.641 | 0.353 | -1.870 |
| 162 | Dataset 2 | -0.523 | 4.81 | -1.361 | 14.308 | 13.333 | 30.133 | 11.246 | 6.981 | -1.016 | 0.321 | -2.128 |
| 163 | Dataset 2 | -0.599 | 4.21 | -1.463 | 12.855 | 14.326 | 58.222 | 8.502 | 7.110 | -1.078 | 0.332 | -2.355 |
| 164 | Dataset 2 | -0.665 | 4.76 | -1.767 | 12.167 | 13.663 | 28.460 | 8.957 | 7.479 | -1.151 | 0.327 | -2.330 |
| 165 | Dataset 2 | -0.624 | 4.71 | -1.777 | 14.344 | 11.545 | 37.405 | 10.159 | 7.146 | -1.201 | 0.265 | -2.352 |
| 166 | Dataset 2 | -0.461 | 6.29 | -2.750 | 6.486 | 9.694 | 31.453 | 8.055 | 5.306 | -1.380 | 0.121 | -2.842 |
| 167 | Dataset 2 | -0.616 | 4.79 | -1.493 | 6.317 | 12.752 | 33.118 | 5.499 | 4.961 | -1.321 | 0.197 | -2.383 |
| 168 | Dataset 2 | -0.385 | 4.25 | -2.269 | 7.689 | 13.893 | 49.190 | 11.833 | 8.720 | -1.483 | 0.080 | -1.975 |
| 169 | Dataset 2 | -0.799 | 5.46 | -1.584 | 13.128 | 11.487 | 16.313 | 11.827 | 4.137 | -1.157 | 0.439 | -2.640 |
| 170 | Dataset 2 | 1.012 | 5.14 | -0.515 | 11.118 | 13.158 | 44.224 | 9.133 | 6.656 | 0.340 | 0.213 | -0.447 |
| 171 | Dataset 2 | -0.269 | 5.04 | -1.144 | 11.668 | 10.777 | 22.719 | 10.121 | 5.962 | -0.463 | 0.640 | -1.870 |
| 172 | Dataset 2 | -0.512 | 5.62 | -1.999 | 13.543 | 9.417 | 29.605 | 13.381 | 4.332 | -1.001 | 0.325 | -2.604 |
| 173 | Dataset 2 | -0.597 | 4.72 | -1.312 | 8.620 | 10.827 | 24.908 | 6.556 | 5.814 | -0.992 | 0.402 | -2.198 |
| 174 | Dataset 2 | -0.187 | 6.40 | -3.629 | 10.967 | 12.095 | 23.361 | 11.915 | 3.248 | -1.137 | 0.112 | -2.341 |
| 175 | Dataset 2 | -0.666 | 5.56 | -2.123 | 8.158 | 12.026 | 22.528 | 6.611 | 5.282 | -1.148 | 0.329 | -2.607 |
| 176 | Dataset 2 | -0.590 | 4.45 | -1.202 | 10.969 | 14.908 | 38.446 | 10.291 | 6.666 | -1.065 | 0.335 | -2.234 |
| 177 | Dataset 2 | -0.449 | 5.27 | -1.886 | 13.067 | 14.302 | 30.897 | 13.021 | 7.553 | -0.807 | 0.438 | -2.210 |
| 178 | Dataset 2 | -0.677 | 4.92 | -1.710 | 7.724 | 10.738 | 30.124 | 7.386 | 4.591 | -1.002 | 0.473 | -2.496 |
| 179 | Dataset 2 | -0.620 | 4.71 | -1.620 | 9.368 | 12.191 | 24.085 | 7.974 | 6.605 | -1.084 | 0.344 | -2.229 |
| 180 | Dataset 2 | -0.440 | 4.59 | -1.183 | 12.776 | 13.848 | 36.265 | 12.523 | 8.329 | -0.733 | 0.509 | -2.045 |
| 181 | Dataset 2 | -0.652 | 4.81 | -1.900 | 9.229 | 10.713 | 29.481 | 8.074 | 5.970 | -1.008 | 0.441 | -2.402 |
| 182 | Dataset 2 | -0.623 | 4.23 | -1.406 | 10.926 | 12.683 | 32.044 | 10.690 | 8.339 | -0.930 | 0.493 | -2.200 |
| 183 | Dataset 2 | -0.524 | 4.66 | -1.662 | 11.050 | 12.886 | 38.058 | 11.300 | 8.618 | -0.906 | 0.415 | -2.246 |
| 184 | Dataset 2 | -0.530 | 4.36 | -1.161 | 11.803 | 12.507 | 30.890 | 9.875 | 7.617 | -0.815 | 0.519 | -2.077 |
| 185 | Dataset 2 | -0.710 | 4.55 | -1.659 | 11.545 | 9.519 | 37.806 | 12.424 | 7.824 | -0.956 | 0.567 | -2.498 |
| 186 | Dataset 2 | -1.018 | 4.30 | -1.583 | 11.327 | 8.799 | 18.225 | 18.485 | 4.432 | -1.376 | 0.438 | -2.529 |
| 187 | Dataset 3 | 0.146 | 7.51 | -2.018 | 10.860 | 19.800 | 17.800 | 8.663 | 3.133 | -1.401 | 0.028 | -2.115 |
| 188 | Dataset 3 | -0.174 | 7.63 | -2.481 | 8.890 | 18.300 | 20.100 | 6.964 | 0.356 | -1.224 | 0.089 | -2.750 |
| 189 | Dataset 3 | -0.229 | 6.54 | -1.639 | 8.330 | 17.300 | 16.900 | 7.880 | 3.350 | -1.321 | 0.081 | -2.025 |
| 190 | Dataset 3 | -0.319 | 7.65 | -3.004 | 7.420 | 19.100 | 19.300 | 7.200 | 3.280 | -1.556 | 0.058 | -3.024 |
| 191 | Dataset 3 | -0.032 | 7.33 | -1.921 | 9.360 | 18.600 | 24.600 | 7.365 | 3.280 | -1.379 | 0.045 | -2.487 |
| 192 | Dataset 3 | -0.398 | 7.37 | -2.639 | 9.350 | 20.100 | 21.300 | 7.346 | 3.400 | -1.856 | 0.035 | -3.046 |
| 193 | Dataset 3 | -0.495 | 7.28 | -3.469 | 4.950 | 19.200 | 21.100 | 6.957 | 4.082 | -1.856 | 0.044 | -3.148 |
| 194 | Dataset 3 | -0.347 | 7.77 | -3.143 | 5.540 | 17.500 | 19.500 | 6.490 | 3.968 | -1.747 | 0.040 | -3.159 |
| 195 | Dataset 3 | -0.013 | 7.30 | -2.229 | 8.820 | 20.700 | 25.700 | 8.604 | 0.313 | -0.587 | 0.267 | -2.431 |
| 196 | Dataset 3 | -0.215 | 7.12 | -2.222 | 8.170 | 18.100 | 23.100 | 7.000 | 3.587 | -1.660 | 0.036 | -2.607 |
| 197 | Dataset 3 | -0.180 | 5.82 | -1.284 | 8.420 | 19.800 | 35.800 | 7.504 | 3.546 | -0.888 | 0.196 | -2.429 |
| 198 | Dataset 3 | -0.328 | 7.17 | -2.494 | 6.720 | 20.600 | 28.500 | 7.658 | 4.038 | -1.622 | 0.051 | -2.991 |
| 199 | Dataset 3 | 0.086 | 5.63 | -0.744 | 7.040 | 16.800 | 21.900 | 8.591 | 6.230 | -0.916 | 0.100 | -1.658 |
| 200 | Dataset 3 | -0.208 | 5.47 | -0.854 | 6.700 | 16.500 | 20.800 | 8.363 | 5.924 | -1.029 | 0.151 | -1.971 |
| 201 | Dataset 3 | -0.009 | 5.68 | -1.000 | 7.620 | 17.000 | 22.700 | 8.513 | 5.110 | -1.002 | 0.102 | -1.830 |
| 202 | Dataset 3 | 0.582 | 5.33 | 0.079 | 6.160 | 12.800 | 14.700 | 7.383 | 4.407 | -0.401 | 0.104 | -0.762 |
| 203 | Dataset 3 | -0.149 | 5.40 | -0.886 | 6.940 | 20.100 | 27.700 | 9.955 | 5.405 | -1.057 | 0.123 | -2.005 |
| 204 | Dataset 3 | 0.493 | 5.76 | -0.494 | 8.180 | 21.100 | 30.100 | 10.185 | 5.620 | -0.701 | 0.064 | -1.305 |
| 205 | Dataset 3 | 0.449 | 5.76 | -0.468 | 12.410 | 21.800 | 31.300 | 11.894 | 5.170 | -0.832 | 0.052 | -1.389 |
| 206 | Dataset 3 | 0.844 | 5.71 | 0.079 | 10.080 | 17.500 | 24.900 | 8.787 | 4.585 | -0.133 | 0.105 | -0.724 |
| 207 | Dataset 3 | 0.143 | 6.53 | -1.284 | 11.260 | 18.800 | 19.900 | 10.905 | 4.534 | -0.737 | 0.132 | -1.553 |
| 208 | Dataset 3 | -0.538 | 5.40 | -1.181 | 12.350 | 20.100 | 22.600 | 10.671 | 8.088 | -1.471 | 0.117 | -2.467 |
| 209 | Dataset 3 | 0.076 | 5.78 | -0.720 | 11.360 | 22.300 | 20.900 | 10.175 | 6.304 | -0.701 | 0.167 | -1.730 |
| 210 | Dataset 3 | 0.053 | 6.05 | -0.959 | 11.860 | 23.600 | 18.500 | 9.785 | 6.700 | -0.850 | 0.125 | -1.876 |
| 211 | Dataset 3 | 0.158 | 6.55 | -1.194 | 12.540 | 23.600 | 18.700 | 10.721 | 5.170 | -0.862 | 0.095 | -1.521 |
| 212 | Dataset 3 | 0.243 | 6.66 | -1.125 | 9.950 | 25.400 | 22.900 | 7.892 | 5.430 | -0.497 | 0.182 | -1.558 |
| 213 | Dataset 3 | 0.400 | 7.33 | -1.677 | 10.460 | 23.900 | 19.000 | 10.240 | 3.956 | -0.659 | 0.087 | -1.658 |
| 214 | Dataset 3 | 0.791 | 6.02 | -0.328 | 10.300 | 27.400 | 26.900 | 8.863 | 5.105 | -0.224 | 0.097 | -0.979 |
| 215 | Dataset 3 | -0.509 | 5.00 | -1.000 | 6.160 | 14.800 | 23.700 | 5.988 | 4.555 | -0.446 | 1.155 | -2.260 |
| 216 | Dataset 3 | -0.444 | 5.52 | -1.222 | 7.100 | 13.000 | 21.100 | 6.150 | 4.094 | -1.321 | 0.133 | -2.338 |
| 217 | Dataset 3 | -0.276 | 4.87 | -0.795 | 7.160 | 14.800 | 25.300 | 6.613 | 5.422 | -0.787 | 0.308 | -1.924 |
| 218 | Dataset 3 | 0.671 | 4.95 | 0.302 | 6.280 | 12.800 | 19.000 | 5.806 | 4.840 | -0.099 | 0.170 | -0.650 |
| 219 | Dataset 3 | 0.732 | 5.03 | 0.380 | 6.040 | 14.000 | 20.700 | 6.347 | 5.005 | -1.010 | 0.018 | -0.616 |
| 220 | Dataset 3 | 0.004 | 5.16 | -0.432 | 5.600 | 13.300 | 21.300 | 5.145 | 4.270 | -0.706 | 0.195 | -1.572 |
| 221 | Dataset 3 | -0.268 | 5.14 | -0.744 | 6.070 | 11.700 | 18.100 | 5.105 | 4.243 | -0.622 | 0.442 | -1.857 |
| 222 | Dataset 3 | 0.225 | 5.86 | -0.698 | 8.110 | 22.100 | 22.900 | 8.609 | 5.365 | -0.555 | 0.166 | -1.597 |
| 223 | Dataset 3 | -0.137 | 6.00 | -1.252 | 12.160 | 23.100 | 24.200 | 10.130 | 7.763 | -1.121 | 0.104 | -2.264 |
| 224 | Dataset 3 | 0.220 | 6.13 | -1.000 | 10.140 | 22.300 | 20.200 | 8.841 | 6.046 | -0.938 | 0.070 | -1.726 |
| 225 | Dataset 3 | 0.802 | 7.15 | -1.309 | 7.280 | 15.000 | 22.900 | 6.440 | 4.555 | -0.809 | 0.024 | -1.042 |
| 226 | Dataset 3 | 0.588 | 7.15 | -1.494 | 7.740 | 15.700 | 22.800 | 6.907 | 0.465 | -1.622 | 0.006 | -1.249 |
| 227 | Dataset 3 | 0.305 | 5.28 | -0.229 | 6.560 | 14.000 | 18.600 | 4.755 | 3.746 | -0.422 | 0.187 | -1.164 |
| 228 | Dataset 3 | 0.161 | 5.67 | -0.553 | 6.060 | 12.000 | 15.600 | 4.498 | 3.397 | -0.339 | 0.316 | -1.407 |
| 229 | Dataset 3 | 0.650 | 5.36 | 0.146 | 6.080 | 11.500 | 16.000 | 4.995 | 4.124 | -0.359 | 0.098 | -0.708 |
| 230 | Dataset 3 | -0.292 | 5.31 | -0.921 | 7.970 | 15.500 | 19.400 | 7.100 | 5.561 | -1.378 | 0.082 | -1.987 |
| 231 | Dataset 3 | -0.553 | 5.38 | -1.236 | 6.910 | 15.300 | 18.400 | 7.046 | 4.965 | -1.497 | 0.114 | -2.357 |
| 232 | Dataset 3 | 0.616 | 6.27 | -0.347 | 10.180 | 25.600 | 13.000 | 10.321 | 4.910 | -1.122 | 0.018 | -1.115 |
| 233 | Dataset 3 | -0.194 | 6.50 | -1.795 | 10.400 | 23.900 | 19.600 | 8.563 | 5.701 | -1.525 | 0.047 | -2.599 |
| 234 | Dataset 3 | -0.301 | 6.43 | -1.585 | 11.520 | 24.600 | 15.300 | 7.400 | 5.922 | -1.321 | 0.096 | -2.577 |
| 235 | Dataset 3 | -0.310 | 6.18 | -1.537 | 12.470 | 24.400 | 22.100 | 8.559 | 6.607 | -1.497 | 0.065 | -2.602 |
| 236 | Dataset 3 | -0.481 | 5.83 | -1.468 | 5.850 | 24.100 | 20.400 | 6.360 | 4.854 | -1.303 | 0.151 | -2.550 |
| 237 | Dataset 3 | -0.125 | 6.96 | -1.795 | 8.570 | 25.100 | 14.900 | 8.312 | 5.458 | -1.038 | 0.122 | -2.100 |
| 238 | Dataset 3 | -0.796 | 5.20 | -1.481 | 9.780 | 22.600 | 19.800 | 10.155 | 9.388 | -1.321 | 0.298 | -2.670 |
| 239 | Dataset 4 | 0.212 | 5.55 | -0.331 | 8.210 | 12.500 | 23.000 | 10.410 | 6.897 | -0.097 | 0.491 | -1.475 |
| 240 | Dataset 4 | 0.332 | 5.56 | -0.230 | 6.930 | 12.600 | 23.100 | 9.989 | 6.289 | -0.032 | 0.433 | -1.314 |
| 241 | Dataset 4 | 0.243 | 5.26 | -0.195 | 7.800 | 12.300 | 22.400 | 9.704 | 6.295 | -0.174 | 0.383 | -1.316 |
| 242 | Dataset 4 | 0.176 | 5.20 | -0.223 | 8.170 | 11.800 | 17.300 | 11.330 | 6.750 | -0.180 | 0.440 | -1.287 |
| 243 | Dataset 4 | 0.025 | 4.87 | -0.243 | 7.390 | 9.470 | 18.800 | 9.945 | 5.757 | 0.182 | 1.434 | -1.417 |
| 244 | Dataset 4 | 0.403 | 5.42 | -0.038 | 8.800 | 12.600 | 15.200 | 12.191 | 6.461 | 0.033 | 0.427 | -1.022 |
| 245 | Dataset 4 | 0.483 | 5.26 | 0.111 | 9.000 | 11.700 | 17.200 | 12.325 | 8.051 | 0.086 | 0.401 | -0.924 |
| 246 | Dataset 4 | 0.382 | 5.37 | -0.022 | 8.350 | 11.600 | 15.300 | 11.620 | 6.783 | -0.398 | 0.166 | -1.036 |
| 247 | Dataset 4 | 0.152 | 5.02 | -0.219 | 8.070 | 12.100 | 19.000 | 10.652 | 6.658 | -0.081 | 0.585 | -1.302 |
| 248 | Dataset 4 | -0.444 | 5.58 | -1.326 | 7.790 | 12.000 | 14.100 | 11.075 | 6.622 | -1.180 | 0.183 | -2.166 |
| 249 | Dataset 4 | 0.660 | 5.78 | 0.113 | 8.830 | 13.200 | 16.100 | 11.519 | 5.856 | 0.199 | 0.346 | -0.845 |
| 250 | Dataset 4 | -0.131 | 4.82 | -0.333 | 6.230 | 7.920 | 15.000 | 7.647 | 5.363 | -0.337 | 0.622 | -1.521 |
| 251 | Dataset 4 | 0.079 | 5.03 | -0.229 | 6.480 | 8.950 | 15.900 | 8.133 | 5.920 | -0.377 | 0.350 | -1.333 |
| 252 | Dataset 4 | 0.258 | 5.14 | -0.147 | 5.550 | 11.600 | 21.500 | 10.863 | 7.904 | -0.018 | 0.530 | -1.244 |
| 253 | Dataset 4 | 0.387 | 5.08 | -0.004 | 8.310 | 12.600 | 20.700 | 11.352 | 7.474 | -0.215 | 0.250 | -1.054 |
| 254 | Dataset 4 | 0.452 | 5.09 | 0.119 | 8.140 | 11.500 | 19.400 | 11.070 | 6.804 | 0.013 | 0.364 | -0.955 |
| 255 | Dataset 4 | 0.199 | 6.07 | -0.409 | 8.380 | 11.700 | 13.300 | 12.637 | 6.558 | -0.018 | 0.608 | -1.550 |
| 256 | Dataset 4 | 0.490 | 4.92 | 0.106 | 8.570 | 11.700 | 19.200 | 11.219 | 7.471 | -0.215 | 0.197 | -0.870 |
| 257 | Dataset 4 | 0.292 | 5.04 | -0.047 | 9.560 | 12.000 | 20.300 | 11.218 | 7.420 | -0.523 | 0.153 | -1.157 |
| 258 | Dataset 4 | 0.375 | 5.54 | -0.172 | 8.260 | 11.800 | 19.700 | 11.253 | 7.390 | -0.041 | 0.384 | -1.191 |
| 259 | Dataset 4 | 0.444 | 5.05 | 0.037 | 5.240 | 11.400 | 22.100 | 10.853 | 7.267 | 0.064 | 0.417 | -0.991 |
| 260 | Dataset 4 | 0.299 | 5.23 | -0.206 | 8.090 | 11.800 | 14.100 | 11.228 | 6.174 | -0.602 | 0.126 | -1.076 |
| 261 | Dataset 4 | -0.328 | 5.09 | -0.989 | 8.590 | 11.600 | 14.800 | 11.772 | 8.027 | -0.854 | 0.298 | -1.845 |
| 262 | Dataset 4 | 0.305 | 5.70 | -0.433 | 9.380 | 12.300 | 15.200 | 11.391 | 7.486 | 0.107 | 0.634 | -1.254 |
| 263 | Dataset 4 | 0.290 | 5.79 | -0.473 | 7.630 | 13.700 | 18.900 | 9.897 | 6.838 | -0.066 | 0.441 | -1.395 |
| 264 | Dataset 4 | 0.312 | 5.74 | -0.372 | 4.170 | 15.100 | 23.900 | 7.267 | 5.127 | -0.347 | 0.220 | -1.427 |
| 265 | Dataset 4 | 0.407 | 7.89 | -2.092 | 6.700 | 15.300 | 23.500 | 6.565 | 5.022 | -0.678 | 0.082 | -2.136 |
| 266 | Dataset 4 | 0.228 | 4.99 | -0.174 | 7.930 | 13.500 | 17.500 | 10.877 | 6.734 | -0.252 | 0.331 | -1.173 |
| 267 | Dataset 4 | 0.459 | 5.28 | -0.125 | 12.700 | 18.700 | 23.600 | 14.128 | 8.942 | -0.824 | 0.052 | -1.075 |
| 268 | Dataset 4 | 0.974 | 6.42 | -0.252 | 11.710 | 20.400 | 30.800 | 12.618 | 6.019 | 0.158 | 0.153 | -1.083 |
| 269 | Dataset 4 | 0.617 | 5.20 | 0.065 | 11.100 | 18.300 | 29.100 | 13.134 | 7.364 | 0.041 | 0.266 | -0.924 |
| 270 | Dataset 4 | 0.428 | 5.72 | -0.344 | 9.100 | 21.200 | 22.000 | 16.727 | 9.666 | -0.252 | 0.209 | -1.258 |
| 271 | Dataset 4 | 0.286 | 5.12 | -0.199 | 5.820 | 15.200 | 24.900 | 12.008 | 6.135 | -0.076 | 0.435 | -1.258 |
| 272 | Dataset 4 | -0.167 | 4.98 | -0.988 | 11.140 | 15.900 | 21.800 | 11.731 | 6.678 | -0.770 | 0.250 | -1.759 |
| 273 | Dataset 4 | -0.658 | 5.18 | -1.518 | 10.350 | 20.700 | 23.600 | 13.116 | 6.875 | -1.319 | 0.218 | -2.556 |
| 274 | Dataset 4 | 1.130 | 5.99 | 0.270 | 14.970 | 29.900 | 32.400 | 17.518 | 9.545 | 0.297 | 0.147 | -0.652 |
| 275 | Dataset 4 | 1.049 | 5.78 | 0.350 | 14.900 | 27.300 | 30.500 | 16.479 | 9.575 | 0.243 | 0.156 | -0.611 |
| 276 | Dataset 4 | 1.009 | 5.78 | 0.333 | 14.500 | 25.400 | 29.500 | 17.034 | 9.823 | 0.176 | 0.147 | -0.654 |
| 277 | Dataset 4 | 0.876 | 6.07 | 0.021 | 13.870 | 26.800 | 29.500 | 16.879 | 8.196 | 0.265 | 0.245 | -0.987 |
| 278 | Dataset 4 | 0.903 | 5.73 | 0.202 | 11.060 | 25.700 | 27.000 | 17.330 | 8.842 | 0.029 | 0.134 | -0.719 |
| 279 | Dataset 4 | 0.584 | 5.88 | -0.249 | 13.610 | 25.800 | 30.800 | 17.241 | 9.350 | -0.155 | 0.182 | -1.287 |
| 280 | Dataset 4 | 0.700 | 5.90 | -0.127 | 15.190 | 24.500 | 31.600 | 17.323 | 8.644 | 0.009 | 0.204 | -1.148 |
| 281 | Dataset 4 | 0.940 | 6.15 | -0.007 | 15.780 | 25.500 | 26.400 | 17.414 | 9.094 | 0.164 | 0.168 | -0.936 |
| 282 | Dataset 4 | 0.220 | 5.75 | -0.440 | 13.200 | 21.500 | 25.700 | 13.078 | 7.896 | -0.367 | 0.259 | -1.623 |
| 283 | Dataset 4 | 0.207 | 5.23 | -0.273 | 14.170 | 23.700 | 23.500 | 14.715 | 9.485 | -0.125 | 0.466 | -1.388 |
| 284 | Dataset 4 | 0.297 | 5.38 | -0.265 | 13.430 | 21.700 | 23.800 | 14.710 | 9.572 | -0.161 | 0.348 | -1.325 |
| 285 | Dataset 4 | 0.362 | 5.48 | -0.323 | 11.690 | 21.200 | 27.800 | 14.458 | 9.044 | -0.310 | 0.213 | -1.341 |
| 286 | Dataset 4 | 0.473 | 5.24 | -0.160 | 11.780 | 19.800 | 26.500 | 13.413 | 8.990 | -0.041 | 0.306 | -1.088 |
| 287 | Dataset 4 | 0.415 | 5.33 | -0.279 | 13.280 | 19.200 | 31.600 | 13.006 | 8.901 | -0.086 | 0.315 | -1.274 |
| 288 | Dataset 4 | 0.336 | 5.39 | -0.376 | 7.270 | 23.400 | 29.600 | 13.377 | 8.582 | -0.215 | 0.281 | -1.363 |
| 289 | Dataset 4 | 0.230 | 5.58 | -0.498 | 13.830 | 23.300 | 26.400 | 13.501 | 8.189 | -0.244 | 0.335 | -1.541 |
| 290 | Dataset 4 | 0.179 | 5.39 | -0.448 | 13.730 | 22.800 | 30.000 | 13.293 | 8.090 | -0.284 | 0.344 | -1.593 |
| 291 | Dataset 4 | 0.064 | 5.83 | -1.063 | 13.550 | 23.900 | 25.700 | 12.763 | 7.858 | -0.432 | 0.319 | -1.893 |
| 292 | Dataset 4 | 0.107 | 5.47 | -0.890 | 12.870 | 23.200 | 28.400 | 12.513 | 8.134 | -0.509 | 0.242 | -1.699 |
| 293 | Dataset 4 | 0.250 | 6.05 | -1.041 | 14.090 | 25.800 | 31.700 | 16.540 | 7.833 | -0.432 | 0.208 | -1.876 |
| 294 | Dataset 4 | 0.276 | 5.50 | -0.537 | 15.330 | 26.000 | 37.200 | 14.228 | 8.079 | -0.301 | 0.265 | -1.618 |
| 295 | Dataset 4 | 0.346 | 5.27 | -0.345 | 12.850 | 22.800 | 24.400 | 11.832 | 8.189 | -0.208 | 0.279 | -1.230 |
| 296 | Dataset 4 | 0.161 | 5.23 | -0.527 | 8.570 | 21.800 | 30.200 | 13.845 | 8.882 | -0.409 | 0.269 | -1.553 |
| 297 | Dataset 4 | -0.310 | 4.99 | -1.107 | 13.620 | 22.053 | 26.600 | 12.454 | 8.976 | -0.921 | 0.245 | -2.052 |
| 298 | Dataset 4 | 0.000 | 5.40 | -1.001 | 11.740 | 24.604 | 28.200 | 12.478 | 8.855 | -0.553 | 0.280 | -1.815 |
| 299 | Dataset 4 | -0.301 | 5.54 | -1.385 | 13.810 | 23.471 | 29.000 | 12.337 | 8.080 | -0.921 | 0.240 | -2.336 |
| 300 | Dataset 4 | 0.045 | 4.96 | -0.371 | 10.770 | 20.634 | 26.100 | 15.244 | 8.805 | -0.398 | 0.360 | -1.551 |
| 301 | Dataset 4 | -0.523 | 5.84 | -1.804 | 11.500 | 23.036 | 25.600 | 14.418 | 7.523 | -1.161 | 0.230 | -2.780 |
| 302 | Dataset 4 | -0.638 | 6.04 | -1.889 | 12.840 | 16.904 | 19.200 | 15.303 | 5.585 | -1.357 | 0.191 | -2.917 |
| 303 | Dataset 4 | 0.378 | 6.38 | -1.055 | 12.050 | 24.876 | 24.100 | 13.513 | 7.768 | -0.921 | 0.050 | -1.807 |
| 304 | Dataset 4 | 0.418 | 5.77 | -0.480 | 10.880 | 22.141 | 25.800 | 17.061 | 7.902 | -0.481 | 0.126 | -1.358 |
| 305 | Dataset 4 | 0.124 | 5.97 | -1.121 | 11.740 | 22.523 | 26.500 | 17.436 | 6.723 | -0.721 | 0.143 | -1.900 |
| 306 | Dataset 4 | -0.721 | 5.46 | -1.802 | 14.110 | 21.722 | 14.500 | 19.927 | 4.245 | -1.721 | 0.100 | -2.521 |
| 307 | Dataset 4 | -0.824 | 4.68 | -1.718 | 12.040 | 26.224 | 17.800 | 13.361 | 2.838 | -1.638 | 0.153 | -2.435 |
| 308 | Dataset 4 | -0.796 | 4.91 | -1.656 | 11.710 | 23.459 | 18.200 | 17.622 | 4.978 | -1.658 | 0.138 | -2.499 |
| 309 | Dataset 4 | -0.143 | 8.03 | -2.968 | 15.820 | 20.471 | 26.000 | 22.032 | 8.299 | -1.108 | 0.108 | -3.218 |
| 310 | Dataset 4 | -0.252 | 8.15 | -3.040 | 10.770 | 14.951 | 14.500 | 18.668 | 6.539 | -1.194 | 0.114 | -3.106 |
| 311 | Dataset 4 | -0.602 | 8.34 | -3.307 | 1.460 | 9.584 | 15.500 | 4.879 | 2.572 | -1.284 | 0.208 | -3.708 |

# Text S1. Machine learning model principles

1. **Ridge**

Ridge regression is a biased estimation regression method specially used for collinear data analysis. Multicollinearity is the presence of high correlation between independent variables [1], which can lead to instability in Ordinary Least Squares estimation, making the model's predictive performance degrade. Ridge regression addresses this problem by adding a regularization term to the loss function that limits the size of the regression coefficients, thereby reducing the impact of multicollinearity on the estimates [2].

1. **Decision Tree**

The principle of Decision Tree (DT) is to divide the data into different categories or regression values to construct a tree-structured model for prediction. The process of building a decision tree is a recursive process that divides the data by selecting the best features and cut points so that the output values of the divided subset are as close as possible to the true values [3].

1. **Support Vector Regression**

Support Vector Regression (SVR) transform the original input vectors into high-dimensional space, making it possible for otherwise nonlinear relationships to become linearly separable in the high-dimensional space [4]. In this space, SVR constructs a linear regression function or hyperplane that keeps most of the data points within the interval and minimizes the prediction error for data points outside the interval.

1. **K-Nearest Neighbors**

The K-Nearest Neighbors (KNN) algorithm calculates the distance of a new sample from other samples in the training set, identifies the K nearest neighbors, and then predicts the value of the new sample based on the values of these neighbors [5-7]. The predicted value of the new sample point is typically the average or weighted average of the values of these K neighbors.

1. **Random Forest**

The Random Forest (RF) [8]is comprised of multiple decision trees, with each tree constructed based on randomly selected samples and features [9]. For a new sample, each decision tree in the forest predicts the value of the sample separately, and the random forest averages or weighted average the predictions of multiple decision trees to obtain the final prediction value [9].

1. **Extremely Randomized Trees**

The construction process of the Extreme Random Trees (ERT) [10] is similar to the RF algorithm. The difference with RF is that at each node, the division threshold of ERT for each feature is also randomly selected, instead of choosing the optimal division threshold. By introducing more randomness, ERT reduces the variance and improves the generalization ability of the model [11].

1. **Gradient Boosting Decision Trees**

Gradient Boosting Decision Trees (GBDT) uses decision trees as the base learner and sums the predictions of a series of trees [12]. Each decision tree is a fitting of the prediction residuals of the previous decision tree combination, which is a modification of the previous model results. Through the integration of multiple weak learners, GBDT usually has high prediction accuracy.

1. **Extreme Gradient Boosting**

Extreme Gradient Boosting (Xgboost) is an ensemble learning method based on Gradient Boosting Tree using decision trees as the basic composition [13]. Xgboost works by training a series of decision trees, each iteration attempting to correct the error of the previous iteration, and ultimately combining these weak learners to form a strong learner.

# Text S2. The code of the Extremely Randomized Trees model

import pandas as pd

import numpy as np

import matplotlib.pyplot as plt

from sklearn.ensemble import ExtraTreesRegressor

from sklearn.model_selection import train_test_split, KFold

from sklearn.metrics import mean_squared_error, r2_score

dataset = pd.read_excel('data311.xlsx')

df = pd.DataFrame(dataset)

print(df.head())

features = df.columns[2:-1]

target = df.columns[-1]

x_train, x_test, y_train, y_test = train_test_split(df[features], df[target], test_size=0.2, random_state=13)

model = ExtraTreesRegressor(n_estimators=120, random_state=42)

k = 10

kf = KFold(n_splits=k, shuffle=True, random_state=42)

mean_squared_error_list, r2_list, rmse_list= [], [], []

for train_index, test_index in kf.split(x_train, y_train):

x_train_fold = x_train.iloc[train_index]

y_train_fold = y_train.iloc[train_index]

x_test_fold = x_train.iloc[test_index]

y_test_fold = y_train.iloc[test_index]

model.fit(x_train_fold, y_train_fold)

y_pred = model.predict(x_test_fold)

mse = mean_squared_error(y_test_fold, y_pred)

mean_squared_error_list.append(mse)

r2 = r2_score(y_test_fold, y_pred)

r2_list.append(r2)

rmse = mse ** 0.5

rmse_list.append(rmse)

print('mse:', mse, 'r2:', r2, 'rmse:', rmse)

average_mse = np.mean(mean_squared_error_list)

print('average mse:', average_mse)

average_rmse = np.mean(rmse_list)

print('average rmse:', average_rmse)

model.fit(x_train, y_train)

y_train_pred = model.predict(x_train)

train_r2 = r2_score(y_train, y_train_pred)

train_mse = mean_squared_error(y_train, y_train_pred)

train_rmse = train_mse ** 0.5

print('=== training ===')

print('MSE:', train_mse)

print('R2:', train_r2)

print('RMSE:', train_rmse)

y_test_pred = model.predict(x_test)

test_mse = mean_squared_error(y_test, y_test_pred)

test_r2 = r2_score(y_test, y_test_pred)

test_rmse = test_mse ** 0.5

def mae(y_test, y_test_pred):

return np.mean(np.abs(y_test - y_test_pred))

mae_value = mae(y_test, y_test_pred)

print('=== test ===')

print('MSE:', test_mse)

print('R2:', test_r2)

print('RMSE:', test_rmse)

print('MAE:', mae_value)

print('=== feature_importance ===')

feature_importance = model.feature_importances_

for i, feature in enumerate(features):

print('{}:{}\n'.format(feature, feature_importance[i]), end='')

feature_names = features

feature_importance_sort = feature_importance.argsort()

plt.subplots(figsize=(8, 6))

plt.barh(range(len(feature_importance)), feature_importance[feature_importance_sort], align='center')

plt.yticks(range(len(feature_importance)), [feature_names[i] for i in feature_importance_sort], fontsize=8)

plt.xlabel('Features Importance')

plt.ylabel('Features Name')

plt.title('Extremely Randomized Trees Regression Feature Importance Evaluation')

plt.show()

FIXED_SEEDS = [8, 13, 17, 19, 42, 63, 71, 77, 82, 90]

results = {

'cv_mse': [], 'cv_r2': [], 'cv_rmse': [],

'test_mse': [], 'test_r2': [], 'test_rmse': [], 'test_mae': []

}

for seed in FIXED_SEEDS:

print(f"\n=== seed {seed} ===")

X_train, X_test, y_train, y_test = train_test_split(

df[features], df[target],

test_size=0.2,

random_state=seed

)

kf = KFold(n_splits=10, shuffle=True, random_state=42)

cv_metrics = {'mse': [], 'r2': [], 'rmse': []}

for train_idx, val_idx in kf.split(X_train):

X_train_fold = X_train.iloc[train_idx]

y_train_fold = y_train.iloc[train_idx]

X_val_fold = X_train.iloc[val_idx]

y_val_fold = y_train.iloc[val_idx]

model = ExtraTreesRegressor(n_estimators=120, random_state=42)

model.fit(X_train_fold, y_train_fold)

y_pred = model.predict(X_val_fold)

cv_metrics['mse'].append(mean_squared_error(y_val_fold, y_pred))

cv_metrics['r2'].append(r2_score(y_val_fold, y_pred))

cv_metrics['rmse'].append(np.sqrt(cv_metrics['mse'][-1]))

results['cv_mse'].append(np.mean(cv_metrics['mse']))

results['cv_r2'].append(np.mean(cv_metrics['r2']))

results['cv_rmse'].append(np.mean(cv_metrics['rmse']))

final_model = ExtraTreesRegressor(n_estimators=120, random_state=42)

final_model.fit(X_train, y_train)

y_test_pred = final_model.predict(X_test)

results['test_mse'].append(mean_squared_error(y_test, y_test_pred))

results['test_r2'].append(r2_score(y_test, y_test_pred))

results['test_rmse'].append(np.sqrt(results['test_mse'][-1]))

results['test_mae'].append(np.mean(np.abs(y_test - y_test_pred)))

def print_statistics(name, values):

print(f"{name}:")

print(f" mean = {np.mean(values):.4f} ± {np.std(values):.4f}")

print(f" range = [{np.min(values):.4f}, {np.max(values):.4f}]")

print(f" median = {np.median(values):.4f}")

print("\n=== cross validation ===")

print_statistics("CV MSE", results['cv_mse'])

print_statistics("CV R²", results['cv_r2'])

print("\n=== test ===")

print_statistics("Test MSE", results['test_mse'])

print_statistics("Test R²", results['test_r2'])

print_statistics("Test RMSE", results['test_rmse'])

print_statistics("Test MAE", results['test_mae'])

plt.figure(figsize=(12, 6))

plt.plot(FIXED_SEEDS, results['test_mse'], 'bo-', label='Test MSE')

plt.plot(FIXED_SEEDS, results['cv_mse'], 'rs--', label='CV MSE')

plt.xlabel('Random Seed')

plt.ylabel('MSE Value')

plt.legend()

plt.grid(True)

plt.xticks(FIXED_SEEDS, rotation=45)

plt.show()

def reliability_analysis(y_true, y_pred, n_bins=10):

bins = np.quantile(y_pred, np.linspace(0, 1, n_bins + 1))

bin_indices = np.digitize(y_pred, bins) - 1

bin_indices = np.clip(bin_indices, 0, n_bins - 1)

bin_actual_means = np.array([y_true[bin_indices == i].mean() for i in range(n_bins)])

bin_pred_means = np.array([y_pred[bin_indices == i].mean() for i in range(n_bins)])

plt.figure(figsize=(12, 6))

plt.subplot(1, 2, 1)

plt.plot(bin_pred_means, bin_actual_means, 's-', color='#1f77b4', label='ERT')

plt.plot([min(y_pred), max(y_pred)], [min(y_pred), max(y_pred)],

'--', color='gray', label='Perfectly calibrated')

plt.xlabel('Mean predicted value', fontsize=10)

plt.ylabel('Mean measured value', fontsize=10)

plt.title('Calibration plots (Reliability Curve)'.format(n_bins))

plt.legend()

plt.grid(alpha=0.3)

plt.subplot(1, 2, 2)

residuals = y_true - y_pred

plt.hist(residuals, bins=30, density=True, alpha=0.6, color='#ff7f0e')

plt.xlabel('Residual error (measured value - predicted value)', fontsize=10)

plt.ylabel(' ', fontsize=10)

plt.title('Residual distribution diagram')

plt.grid(alpha=0.3)

plt.tight_layout()

plt.savefig('reliability.jpg')

plt.show()

print("=" * 40)

print(f" mean residuals: {np.mean(residuals):.4f}")

print(f" std residuals: {np.std(residuals):.4f}")

print(f" median abs residuals: {np.median(np.abs(residuals)):.4f}")

print("\n=== reliability analysis ===")

reliability_analysis(y_test.values, y_test_pred)

print('\n=== test ===')

print('MSE:', test_mse)

print('R2:', test_r2)

print('RMSE:', test_rmse)

y_test_pred_all = np.stack([tree.predict(x_test) for tree in model.estimators_])

lower_bound = np.percentile(y_test_pred_all, 5, axis=0)

upper_bound = np.percentile(y_test_pred_all, 95, axis=0)

mean_pred = y_test_pred_all.mean(axis=0)

sorted_idx = np.argsort(y_test.values)

x_axis = np.arange(len(sorted_idx))

plt.figure(figsize=(12, 6))

plt.fill_between(x_axis,

lower_bound[sorted_idx],

upper_bound[sorted_idx],

color="skyblue",

alpha=0.4,

label="90% Confidence Band")

plt.plot(x_axis, mean_pred[sorted_idx],

"s-", color="tomato",

markersize=4, linewidth=1,

label="Predicted Mean")

plt.plot(x_axis, y_test.values[sorted_idx],

"o-", color="navy",

markersize=4, linewidth=1,

label="Measured Values")

plt.xticks(x_axis[::10], rotation=45)

plt.xlabel("Ordered Samples", fontsize=10)

plt.ylabel("Target Value", fontsize=10)

plt.title("Uncertainty Analysis with 90% Prediction Interval", fontsize=12)

plt.legend(loc="upper left")

plt.grid(alpha=0.3)

plt.tight_layout()

plt.savefig("prediction_interval.jpg")

plt.show()

# Reference

[1] C.B. García, J. García, M.M. López Martín, R. Salmerón, Collinearity: revisiting the variance inflation factor in ridge regression, Journal of Applied Statistics 42 (2014) 648-661.

[2] M.H.D.M. Ribeiro, R.G. da Silva, V.C. Mariani, L.d.S. Coelho, Short-term forecasting COVID-19 cumulative confirmed cases: Perspectives for Brazil, Chaos, Solitons & Fractals 135 (2020).

[3] L. Rokach, Decision forest: Twenty years of research, Information Fusion 27 (2016) 111-125.

[4] M. Awad, R. Khanna. Support Vector Regression [M]. Efficient Learning Machines: Theories, Concepts, and Applications for Engineers and System Designers. Berkeley, CA; Apress. 2015: 67-80.

[5] R.K. Halder, M.N. Uddin, M.A. Uddin, S. Aryal, A. Khraisat, Enhancing K-nearest neighbor algorithm: a comprehensive review and performance analysis of modifications, Journal of Big Data 11 (2024).

[6] K. Taunk, S. De, S. Verma, A. Swetapadma, A Brief Review of Nearest Neighbor Algorithm for Learning and Classification, International Conference on Intelligent Computing and Control Systems (ICCS) (2019) 1255-1260.

[7] Z. Zhang, Introduction to machine learning: k-nearest neighbors, Annals of Translational Medicine 4 (2016) 218-218.

[8] L. Breiman, Random Forests, Machine Learning (2001) 5–32.

[9] M. Schonlau, R.Y. Zou, The random forest algorithm for statistical learning, Stata Journal 20 (2020).

[10] P. Geurts, D. Ernst, L. Wehenkel, Extremely randomized trees, Machine Learning 63 (2006) 3-42.

[11] F. Kazemi, N. Asgarkhani, R. Jankowski, Machine learning-based seismic fragility and seismic vulnerability assessment of reinforced concrete structures, Soil Dynamics and Earthquake Engineering 166 (2023).

[12] W. Liang, S. Luo, G. Zhao, H. Wu, Predicting Hard Rock Pillar Stability Using GBDT, XGBoost, and LightGBM Algorithms, Mathematics 8 (2020).

[13] A. Ibrahem Ahmed Osman, A. Najah Ahmed, M.F. Chow, Y. Feng Huang, A. El-Shafie, Extreme gradient boosting (Xgboost) model to predict the groundwater levels in Selangor Malaysia, Ain Shams Engineering Journal 12 (2021) 1545-1556.
